# Cardiomyocyte programmed cell death in dilated cardiomyopathy: molecular crosstalk and therapeutic implications

**DOI:** 10.3389/fcell.2026.1815510

**Published:** 2026-06-02

**Authors:** Yueqing Qiu, Zhenyi Chen

**Affiliations:** 1 Basic Medical College of Yunnan University of Chinese Medicine, Kunming, Yunan, China; 2 Second School of Clinical Medicine, Henan University of Chinese Medicine, Zhengzhou, Henan, China; 3 Department of Cardiovascular Medicine, Second Affiliated Hospital of Henan University of Chinese Medicine, Zhengzhou, Henan, China

**Keywords:** programmed cell death, apoptosis, necroptosis, pyroptosis, autophagy, ferroptosis, cuproptosis, dilated cardiomyopathy

## Abstract

Progressive cardiomyocyte loss constitutes the pathological hallmark of dilated cardiomyopathy (DCM), a process driven by an intricate network of programmed cell death (PCD) pathways. This review systematically examines the molecular underpinnings and reciprocal crosstalk among six principal PCD modalities implicated in DCM: apoptosis, necroptosis, pyroptosis, autophagy, ferroptosis, and cuproptosis. Apoptosis is triggered by genetic defects—most notably titin (*TTN)* truncating variants—epigenetic dysregulation, and endoplasmic reticulum stress, converging on the activation of both intrinsic and extrinsic caspase cascades. Necroptosis is distinguished by the aberrant nuclear accumulation of phosphorylated MLKL, particularly at the pThr^357^ residue, which selectively exacerbates cardiac dysfunction; upstream events governing this pathway include desmoplakin deficiency, PGC-1α downregulation, and TAB2 loss. Pyroptosis, orchestrated by the NLRP3–GSDMD–IL-1β axis, is robustly activated in the failing myocardium—often exceeding the magnitude of concurrent apoptosis—and propagates a pro-inflammatory milieu through the release of potent cytokines. Autophagy exhibits a pronounced bidirectional effect in DCM: while physiological autophagic flux exerts cardioprotective actions, impaired flux or hyperactivation accelerates cell demise. Ferroptosis is driven by the collapse of the System Xc^−^–GPX4 antioxidant axis and dysregulation of the FSP1 shunt, culminating in lethal lipid peroxidation; this process is subject to upstream regulation by the Hippo–Mst1–NFS1 cascade and m^6^A epigenetic modifications, and it engages in a vicious cycle with downstream inflammation and fibrosis. Cuproptosis ensues from FDX1-mediated copper binding to lipoylated tricarboxylic acid cycle enzymes, precipitating the loss of iron–sulfur cluster proteins and proteotoxic stress; bioinformatic analyses further implicate its interplay with immune infiltration. These PCD pathways do not operate in isolation but rather form a tightly woven molecular crosstalk network *via* shared signaling nodes—including RIPK1, reactive oxygen species (ROS), mitochondrial machinery, and the caspase family—and functional compensation, thereby collectively dictating cardiomyocyte fate and disease trajectory. In light of this network-centric framework, therapeutic strategies that target critical hubs such as GSDMD, GPX4, or RIPK1, or leverage pathway interdependencies for combinatorial intervention, hold considerable promise for disrupting maladaptive feed-forward loops and preserving myocardial integrity. Future investigations should prioritize the delineation of patient-specific PCD network topologies in DCM to pave the way for precision-based therapeutic targeting.

## 1 Introduction

Dilated cardiomyopathy (DCM) is a heterogeneous disorder marked by progressive ventricular dilation, wall thinning, and systolic dysfunction. The myocardium appears pale, flaccid, and fibrotic. Five-year mortality remains high, and therapeutic options are limited, underscoring the substantial burden of DCM (Heymans et al., 2023; Anonymous, 2019).

The Nomenclature Committee on Cell Death (NCCD, 2018) classifies cell death as either accidental cell death (ACD) or programmed cell death (PCD). ACD is an unregulated, passive process triggered by direct physical, chemical, or biological injury (Nagata and Tanaka, 2017). PCD, conversely, is governed by precise molecular mechanisms, exhibits distinct biochemical and morphological features, and is amenable to pharmacological or genetic intervention. This druggability renders PCD pathways attractive therapeutic nodes (Qi et al., 2022).

Narula et al. (1996) first detected apoptosis in myocardial tissue from patients with end-stage heart failure secondary to DCM, establishing PCD as a contributor to DCM pathogenesis. Subsequent work has implicated at least five additional PCD modalities in disease initiation and progression: necroptosis, pyroptosis, ferroptosis, autophagy, and cuproptosis (Del Re et al., 2019). Although each pathway relies on a distinct signaling cascade, they share common triggers (oxidative stress and mitochondrial damage), molecular components (B-cell lymphoma 2 (Bcl-2) family proteins and RIP kinases), and regulatory nodes, thereby forming a complex, interconnected network.

A critical limitation, however, is that most evidence derives from rodent DCM models and *in vitro* systems. The relative contribution and temporal hierarchy of distinct PCD pathways in human DCM myocardium lack systematic clinical validation. Whether emerging cell death subroutines, including mitotic death, immunogenic cell death, and PARP1-dependent parthanatos, participate in DCM pathogenesis remains unknown. This review dissects the molecular mechanisms and reciprocal crosstalk among the six core PCD pathways driving cardiomyocyte loss in DCM, aiming to provide a mechanistic framework for developing targeted interventions. Table 1 summarizes the morphological features, effector molecules, and immunological characteristics of different types of programmed cell death.

**TABLE 1:** Morphological characteristics, effector molecules, and immunological characteristics of different types of PCD.

RCD type	Morphological characteristics	Effector molecule	Immunological characteristics
Apoptosis	Cells shrink and undergo pyknosis, forming apoptotic bodies that contain cellular components	Bcl-2, caspase, BIM, Bcl-xL	Not related to inflammation
Necroptosis	Cells swell, lyse, and release pro-inflammatory factors	PIPK1, PIPK3, MLKL	Pro-inflammatory
Autophagy	Formation of phagosomes, autophagosomes, and autolysosomes	LC3-II, AGT9, ROS	Not related to inflammation
Pyroptosis	The cell membrane ruptures, and the cellular contents are released	Caspases, GSDMD	Pro-inflammatory
Ferroptosis	The mitochondrial cristae decrease (or disappear), and the outer mitochondrial membrane shrinks	Fe^2+^, GSH, GPX4, ROS	Pro-inflammatory
Cuproptosis	Mitochondria shrink, and the mitochondrial membrane ruptures	Cu^+^, FDX1, acylated protein	Pro-inflammatory

## 2 Apoptosis in DCM

Apoptosis is a genetically orchestrated, autonomous cell death program. Morphologically, it features cell shrinkage, nuclear condensation, and the formation of membrane-bound apoptotic bodies that are rapidly cleared by phagocytes (Letai, 2022). Because apoptotic cells actively suppress inflammatory signaling and are swiftly removed, this process typically avoids eliciting an inflammatory response. Execution of the apoptotic program depends on the activation of the cysteine-aspartic protease (caspase) family via two principal routes: the extrinsic and intrinsic pathways (Yuan and Ofengeim, 2024). The extrinsic pathway is initiated by the binding of cognate ligands (tumor necrosis factor, TNF; Fas ligand, FasL; and TNF-related apoptosis-inducing ligand, TRAIL) to plasma membrane death receptors (TNF receptor 1, TNFR1; Fas; death receptor 4, DR4; and death receptor 5, DR5). The intrinsic pathway, in contrast, responds to intracellular stress signals such as growth factor withdrawal, mitochondrial damage, or DNA lesions. Central to intrinsic apoptosis is the regulatory hub formed by the Bcl-2 protein family. Upon sensing stress, BH3-only proteins neutralize anti-apoptotic members (Bcl-2, Bcl-xL, myeloid cell leukemia 1, and MCL-1) while concurrently activating the pro-apoptotic effectors BAX and BAK. Activated BAX and BAK oligomerize within the mitochondrial outer membrane, inducing mitochondrial outer membrane permeabilization (MOMP). MOMP facilitates the release of cytochrome c and the second mitochondria-derived activator of caspase/direct inhibitor of apoptosis-binding protein with low pI (SMAC/DIABLO) into the cytosol. Cytochrome c associates with apoptotic protease-activating factor 1 (APAF1) and pro-caspase-9 to assemble the apoptosome, which activates caspase-9 and subsequently cleaves the executioner caspases, caspase-3 and caspase-7, driving cellular disassembly. Simultaneously, SMAC/DIABLO antagonizes inhibitor of apoptosis proteins (IAPs), thereby relieving IAP-mediated caspase inhibition and establishing a positive feedback amplification loop (Del Re et al., 2019; Sanz et al., 2023; Newton et al., 2024).

### 2.1 Genetic regulation of apoptosis

Approximately one-third of DCM cases are linked to gene mutations. Over 50 pathogenic genes have been identified, with sarcomeric protein variants accounting for 35%–40% of inherited DCM (Paldino et al., 2018). Titin (*TTN*) truncating variants represent the predominant genetic cause, underlying roughly 25% of familial DCM cases (Mcnally and Mestroni, 2017). Zhou et al. (2017) employed heterozygous *TTN* truncation mice to model DCM and observed marked apoptotic activation accompanied by downregulation of immediate early response 3 (*IER3*). Mechanistically, nuclear factor kappa B (NF-κB) undergoes nuclear translocation, binds the *IER3* promoter, and drives its transcription. The translated IER3 protein then shuttles into the nucleus, where it directly engages promoter regions of apoptosis-related genes to upregulate anti-apoptotic factors, including Bcl-2, Bcl-2 like 1 (Bcl-2L1), and AKT serine/threonine kinase 1 (AKT1). This establishes a protective NF-κB–IER3–anti-apoptotic transcriptional axis.

These findings, however, have yet to be independently validated in myocardial samples from patients with DCM harboring *TTN* truncating variants (*TTN*tvs). The expression dynamics and regulatory weight of IER3 in human DCM remain undefined. Moreover, direct molecular evidence linking *TTN*tvs to upstream NF-κB activation is lacking, a gap that constrains the clinical translation of this axis as a therapeutic target.

### 2.2 Epigenetic regulation of apoptosis

#### 2.2.1 Role of histone deacetylases in cardiomyocyte apoptosis in DCM

Histone deacetylases (HDACs) are core epigenetic enzymes that remove acetyl groups from lysine residues on histones and non-histone proteins, thereby modulating chromatin architecture and gene transcription (Yang and Seto, 2008). The mammalian HDAC family comprises 18 isoforms grouped into four classes: class I (HDAC1, HDAC2, HDAC3, and HDAC8), class II (IIa: HDAC4, HDAC5, HDAC7, and HDAC9; IIb: HDAC6 and HDAC10), class III (sirtuins), and class IV (HDAC11) (Haberland et al., 2009).

Class I HDAC1 and HDAC2 exhibit functionally redundant and indispensable roles in the heart. Cardiomyocyte-specific dual deletion of HDAC1 and HDAC2 in mice precipitates neonatal arrhythmias, a DCM phenotype, and death within 2 weeks. Myocardial TUNEL-positive cells increase approximately three-fold, indicative of marked apoptotic activation (Montgomery et al., 2007). Gene expression profiling reveals aberrant upregulation of genes encoding skeletal muscle-specific contractile proteins, such as troponin I type 2 (Tnni2), and calcium channel constituents (Montgomery et al., 2007). HDAC1 and HDAC2, thus, maintain the terminally differentiated cardiomyocyte state and normal cardiac function by suppressing ectopic expression of non-myocardial lineage genes. Their loss directly triggers apoptosis and DCM.

Class IIb HDAC6 is upregulated in DCM and serves as a regulatory node linking apoptosis and autophagy. HDAC6 knockdown confers protection through a dual mechanism: it enhances autophagic clearance of damaged organelles while concurrently inhibiting NLR family pyrin domain containing 3 (NLRP3) inflammasome activation, thereby reducing apoptosis. Overexpression of NLRP3 abrogates this protective effect, confirming that HDAC6 regulates apoptosis *via* NLRP3 (Pang et al., 2023). In BAG3-knockout DCM mice, the HDAC6-specific inhibitor TYA-018 alleviates sarcomeric injury, improves mitochondrial function, reduces apoptosis, and elevates left ventricular ejection fraction (LVEF), further substantiating its therapeutic potential (Yang et al., 2022).

Class IIa HDAC5 has been identified as a critical regulator in *TTN*tv-associated DCM. Knockdown of HDAC5 reverses cardiac dysfunction and profibrotic gene expression induced by TTN deficiency (Hasan et al., 2025).

The therapeutic promise of HDAC inhibitors (HDACis) in DCM has been preliminarily validated. Class I/II HDACis improve acetylation status and energetic profiles in myocardial mesenchymal stem cells derived from patients with DCM, activate cardiac transcription factors (NK2 homeobox 5, NKX2-5; homeodomain-only protein, HOPX; GATA binding protein 4, GATA4; and myocyte enhancer factor 2C, Mef2C), and upregulate myocardial structural protein expression (Mikšiūnas et al., 2024). The class IIa-selective inhibitor TMP-195 reverses gene expression dysregulation caused by TTN deficiency (Hasan et al., 2025).

Nonetheless, the isoform-specific contributions of distinct HDACs to apoptosis in DCM and their crosstalk with other PCD pathways, such as ferroptosis, await further elucidation. The clinical translational prospects of isoform-selective HDAC inhibitors require further rigorous evaluation.

#### 2.2.2 DNA methylation

DNA methylation, the addition of a methyl group to the cytosine base of cytosine–phosphate–guanine (CpG) dinucleotides catalyzed by DNA methyltransferases, is a dynamic and reversible process that represents a potential target for disease risk prediction and intervention (Sergeeva et al., 2023).

Widespread DNA methylation abnormalities characterize the myocardium of patients with DCM. Genome-wide analyses reveal that differentially methylated regions are enriched in pathways relevant to cardiac disease and that aberrant methylation correlates significantly with altered expression of lymphocyte antigen 75 (LY75) and adenosine A2a receptor (ADORA2A) (Haas et al., 2013). Global alterations in 5-methylcytosine (5-mC) and 5-hydroxymethylcytosine (5-hmC) within left ventricular tissue further suggest a role for these modifications in DCM pathogenesis (Tabish et al., 2019). An epigenome-wide association study identified 194 DCM-associated CpG sites in left ventricular tissue; 32 of these sites significantly influence the expression of adjacent genes involved in cell death, cell cycle progression, and metabolic regulation (Tan et al., 2025). Integrated analysis of atrial tissue uncovered over 23,000 differentially methylated regions. Promoter hypomethylation of five key genes, including natriuretic peptide A (*NPPA*), natriuretic peptide B (*NPPB*), and actinin alpha 2 (*ACTN2*), was accompanied by their transcriptional upregulation, implicating epigenetic dysregulation directly in myocardial stress responses and structural gene regulation (Guo Z. et al., 2025). Integrative bioinformatics further identified eight aberrantly methylated, differentially expressed genes, and subsequent validation in DCM mouse hearts confirmed elevated expression of solute carrier family 16 member 9 (*SLC16A9*), synuclein alpha (*SNCA*), phosphodiesterase 5A (*PDE5A*), fibronectin type III domain containing 1 (*FNDC1*), and HtrA serine peptidase 1 (*HTRA1*) (Li N. et al., 2024). Nonetheless, most of these findings remain correlative, linking altered methylation to changes in gene expression. Functional causal evidence defining the precise transcriptional networks through which DNA methylation directly regulates apoptotic executioners is lacking.

Lamin A/C (*LMNA*)-related DCM provides a comparatively well-defined mechanistic paradigm. In cardiomyocytes harboring *LMNA* pathogenic variants, lamin-associated domains undergo redistribution, accompanied by pronounced shifts in CpG methylation. These changes are associated with the differential expression of approximately 4,500 coding genes and 800 long non-coding RNAs (lncRNAs), with the tumor protein p53 (P53) pathway exhibiting the most significant dysregulation (Cheedipudi et al., 2019). Subsequent work confirmed that pathogenic activation of the E2F transcription factor (E2F)/P53 axis directly induces cardiomyocyte apoptosis, fibrosis, and cardiac dysfunction (Chen et al., 2019). These observations delineate a mechanistic cascade extending from chromatin structural reorganization to altered methylation and culminating in apoptotic pathway activation. Whether a similar cascade operates in sporadic DCM unrelated to *LMNA* pathogenic variants remains to be established.

Functional evidence for a DNA methylation-apoptosis regulatory axis has also emerged from animal models. Selenium supplementation inhibits DNA methyltransferase 2 (DNMT2)-mediated promoter methylation of glutathione peroxidase 1 (GPX1), thereby upregulating GPX1 expression, suppressing reactive oxygen species (ROS)-mediated cardiomyocyte apoptosis, and improving cardiac function in heart failure (Zhu et al., 2022). Separately, PIWI-interacting RNA-6426 (piRNA-6426) promotes the enrichment of DNA methyltransferase 3B (DNMT3B) at the sterol O-acyltransferase 1 (*SOAT1*) promoter, modulating its methylation and consequently inhibiting cardiomyocyte apoptosis, inflammation, and oxidative stress (Zhong et al., 2022). The therapeutic efficacy of these interventions, specifically within the context of DCM, awaits clarification. Moreover, although reciprocal regulation between DNA methylation and non-coding RNAs participates in endothelial-to-mesenchymal transition in DCM (Wang E. et al., 2023), the functional points of convergence with established microRNA (miRNA), long non-coding RNA (lncRNA), and circular RNA (circRNA) regulatory axes remain largely unresolved.

In summary, investigations into DNA methylation in the regulation of cardiomyocyte apoptosis in DCM reveal several defining features. First, widespread methylation aberrations are confirmed, yet overlap among candidate genes across studies is limited and functional validation remains sparse. Second, *LMNA*-related DCM offers a mechanistic paradigm, but its broader applicability is uncertain. Third, the hierarchical regulatory relationships between DNA methylation and non-coding RNAs, and their integrated effects on apoptotic pathways, constitute a critical gap in current knowledge.

#### 2.2.3 Non-coding RNAs

##### 2.2.3.1 MicroRNA regulatory axes

MicroRNA-185-5p (miR-185-5p) is upregulated in failing myocardium and correlates positively with transforming growth factor beta 1 (TGF-β1) and type I collagen levels, implicating this miRNA in profibrotic signaling (Lin et al., 2022). It also induces mitochondrial dysfunction and cardiac hypertrophy (Wang T. et al., 2023). Notably, cardiac-targeted liposomal delivery of a miR-185-5p inhibitor suppresses markers of both apoptosis and cuproptosis while restoring Bcl-2 expression *in vitro* and *in vivo*, suggesting that miR-185-5p concurrently regulates two distinct PCD pathways (Xu et al., 2025). This conclusion, however, relies on indirect inference from altered marker abundance rather than direct functional validation of apoptotic executioners (caspase activity) or cuproptotic effectors (dihydrolipoamide S-acetyltransferase (DLAT) oligomerization). Moreover, the clinical translatability of the liposomal delivery system awaits rigorous assessment.

In models of alcoholic cardiomyopathy (ACM), miR-186-5p targets the 3′untranslated region (3′UTR) of X-linked inhibitor of apoptosis (*XIAP*) messenger RNA, suppressing XIAP expression and thereby relieving XIAP-mediated inhibition of caspases to promote cardiomyocyte apoptosis (Liu and Yu, 2019). Although this axis is well validated in AC16 cells, ACM represents only a subset of DCM etiologies. Whether this mechanism extends to primary DCM requires further clarification.

##### 2.2.3.2 CircRNA and lncRNA regulatory networks

Circular RNAs are integral components of gene regulatory networks that help maintain normal cardiac function and contribute to heart disease pathogenesis (Parsanathan et al., 2025). RNA binding proteins (RBPs), which serve as principal regulators of circRNA biogenesis, have been implicated in DCM when deficient (Fathima et al., 2025). The RBP RNA binding motif protein 24 (RBM24) promotes the circularization of circ23679 through phosphorylation dependent splicing regulation. circ23679 functions as a molecular sponge for microRNA-15b-5p (miR-15b-5p). Loss of circ23679 induces cardiomyocyte apoptosis, whereas its overexpression ameliorates the heart failure phenotype in mice (Liu et al., 2025). This work delineates a linear RBM24–circ23679–miR-15b-5p regulatory pathway. Nonetheless, a systematic map of the spatiotemporal expression patterns of circRNAs *in vivo* and their cooperative regulation with other RBPs remains absent.

Long non-coding RNAs also play critical roles in the initiation and progression of cardiac disease (Robinson and Port, 2022). In a doxorubicin (DOX)-induced DCM model, long intergenic non-protein coding RNA 339 (*LINC00339*) is markedly upregulated and directly binds miR-484 to modulate collagen synthesis (Li et al., 2018). A separate axis, lncRNA H19 and miR-675, promotes cardiomyocyte apoptosis by targeting proliferation-associated 2G4 (*PA2G4*) (Zhang et al., 2017). Both axes were identified in DOX toxicity models. Pathologically, DOX-induced DCM differs fundamentally from primary DCM; the former centers on mitochondrial toxicity, whereas the latter involves sarcomeric gene variants and chronic neurohumoral activation. Consequently, the expression profiles and functional contributions of these lncRNA axes in non-toxic DCM await independent validation.

In summary, non-coding RNAs participate in the regulation of cardiomyocyte apoptosis in DCM through competing endogenous RNA (ceRNA) networks, post-transcriptional control, and other mechanisms. Current evidence, however, remains fragmented. Most findings originate from single models or cell lines and lack cross-model corroboration. The hierarchical relationships and synergistic regulatory networks among distinct non-coding RNA species have yet to be integrated. Moreover, the direct role of classical epigenetic modifications, particularly histone modifications, in governing cardiomyocyte apoptosis in DCM is poorly defined. Existing investigations have largely centered on correlative analyses of DNA methylation patterns or the global cardioprotective effects of HDAC inhibitors. The specific transcriptional networks through which histone modifications directly modulate apoptotic signaling, together with the functional weighting of these pathways across different stages of DCM, remain unclear.

### 2.3 Endoplasmic reticulum stress-associated apoptotic pathways in DCM

Accumulation of unfolded proteins within the endoplasmic reticulum (ER) triggers ER stress and activates the unfolded protein response (UPR). Under physiological conditions the UPR preserves ER homeostasis. Persistent ER stress driven by pathological insults such as oxidative stress, ischemia, or hypoxia, however, can precipitate cardiomyopathy (Iurlaro and Muñoz-Pinedo, 2016; Ren et al., 2021). The UPR is governed by three principal signaling axes: protein kinase R like ER kinase (PERK), activating transcription factor 6 (ATF6), and inositol-requiring enzyme 1 (IRE1). The transcription factor C/EBP homologous protein (CHOP) serves as the central executioner of the ER stress apoptotic pathway. CHOP drives apoptosis by downregulating anti-apoptotic proteins, including Bcl-2 and Bcl-xL, while concurrently upregulating pro-apoptotic factors such as Bcl-2 interacting mediator of cell death (BIM) and growth arrest and DNA damage inducible protein 34 (GADD34) (Al-Yacoub et al., 2021). Sustained ER stress further potentiates IRE1α and ATF6 signaling, establishing a positive feedback loop that amplifies CHOP expression (Ren et al., 2021).

Non-coding RNAs also participate in ER stress regulation. Expression of the long non-coding RNA AC061961.2 is reduced in the myocardium of patients with DCM (Qiu et al., 2019). This lncRNA activates WNT/β-catenin signaling, promoting the binding of the β-catenin/TCF transcription factor complex to the glucose-regulated protein 78 (*GRP78*) promoter and enhancing its transcription. The resultant improvement in protein folding attenuates ER stress and suppresses cardiomyocyte apoptosis (Qiu et al., 2021).

Micronutrient deficiency can similarly induce cardiomyocyte apoptosis via ER stress. Keshan disease, an endemic DCM confined to low-selenium regions, exemplifies this phenomenon. Affected individuals exhibit markedly elevated rates of cardiomyocyte apoptosis (Zheng et al., 2021). The chemical chaperone 4-phenylbutyric acid (4-PBA) alleviates cardiomyocyte apoptosis in selenium-deficient rats by inhibiting ER stress signaling. Concurrently, impaired glutathione peroxidase (GPX) activity resulting from selenium deficiency compromises hydrogen peroxide (H_2_O_2_) detoxification, thereby implicating oxidative stress as a contributing factor (Wang et al., 2013). Core apoptotic regulators, including tumor protein p53 (p53), BAX, Bcl-2, and Bcl-xL, have been documented to participate in selenium deficiency-induced cardiomyocyte apoptosis (Shan et al., 2015). Notably, whether the ER stress-apoptosis axis identified in Keshan disease, a geographically restricted DCM subtype, extends to idiopathic or genetic forms of DCM lacks comparative evidence.

In summary, ER stress-mediated cardiomyocyte apoptosis represents a significant pathological mechanism in DCM. Its sustained activation can be fueled by non-coding RNA dysregulation, micronutrient deficiency, and oxidative stress. Whether synergistic or antagonistic interactions exist among the three delineated regulatory routes—the lncRNA–ER stress axis, the selenium deficiency–ER stress axis, and the canonical UPR signaling axis—and their relative contributions across distinct DCM etiological subtypes remain critical unresolved questions.

### 2.4 Autophagy-related apoptosis in DCM

Cytochrome P450 family 2 subfamily E member 1 (CYP2E1), a prominent member of the cytochrome P450 enzyme superfamily, catalyzes the metabolism of diverse endogenous and exogenous substrates (Haduch et al., 2023). Under myocardial stress, CYP2E1 is overexpressed and exacerbates oxidative stress and cardiomyocyte apoptosis through increased generation of ROS (Lu et al., 2012). In cardiac troponin T R141W (cTnT ^R141W^) transgenic DCM mice, either knockdown of CYP2E1 or treatment with the competitive inhibitor diallyl sulfide (DAS) attenuates mitochondria-dependent apoptosis and improves cardiac function (Pang et al., 2021). Mechanistically, the proto-oncogene product Myc directly binds the *CYP2E1* promoter to drive its transcription, and Myc itself is subject to coordinated regulation by extracellular signal-regulated kinase 1 and 2 (ERK1/2) and phosphatidylinositol 3-kinase (PI3K)/protein kinase B (AKT) signaling (Guan et al., 2019). These findings, however, derive exclusively from genetic DCM models; their translational relevance to idiopathic DCM awaits assessment. In stark contrast, cytochrome P450 family two subfamily J member 2 (CYP2J2) attenuates ethanol-induced apoptosis by generating epoxyeicosatrienoic acids (EETs), which activate AMP-activated protein kinase (AMPK)/mechanistic target of rapamycin (mTOR) signaling to induce protective autophagy (Zhou et al., 2018). The functional antagonism exhibited by two distinct cytochrome P450 members underscores the importance of isoform-specific regulation. Whether CYP2E1 directly modulates autophagic flux in DCM, however, remains an open question.

### 2.5 Apoptosis in DOX-induced DCM

In DOX-induced DCM models, cardiomyocyte apoptosis arises from the coordinated dysregulation of multiple signaling cascades.

#### 2.5.1 Energy metabolism and autophagy axis

DOX administration results in malondialdehyde (MDA) accumulation and suppressed superoxide dismutase (SOD) and glutathione (GSH) activity. Metformin activates AMPK, inhibits mTOR, reduces cleaved caspase-3 abundance, and elevates the microtubule-associated protein 1 light chain 3 II (LC3II) to LC3I ratio, implicating autophagy activation in the attenuation of oxidative stress and apoptosis (Zhang S. et al., 2023). Conversely, cardiac-specific *mTOR* knockout mice rapidly develop DCM. mTOR deficiency triggers cardiomyocyte apoptosis via the ankyrin repeat domain 1 (ANKRD1)/c-Jun N-terminal kinase (JNK)/p53 pathway and, by abolishing upstream regulation of hypoxia-inducible factor 1 alpha (HIF-1α), deprives the myocardium of hypoxic adaptation, thereby exacerbating injury (Mazelin et al., 2016). These observations indicate that mTOR exerts a dual function in apoptotic regulation; its net effect likely depends on the nature of the stress and the disease stage.

#### 2.5.2 Mitochondrial function axis

DOX treatment markedly downregulates cytochrome c oxidase subunit 5A (COX5A) in mouse hearts and H9C2 cardiomyocytes. Overexpression of COX5A suppresses DOX-induced elevation of superoxide dismutase 2 (SOD2) and cleaved caspase-3, ameliorates oxidative stress and mitochondrial dysfunction, and attenuates cardiomyocyte apoptosis. Furthermore, DOX reduces phosphorylation of AKT at threonine 308 (Thr308) and serine 473 (Ser473), an effect reversed by COX5A upregulation (Zhang P. et al., 2023).

#### 2.5.3 Inflammatory signaling axis

In DOX-induced DCM mice, cluster of differentiation 47 (CD47) expression is markedly upregulated. A CD47-neutralizing antibody (aCD47) ameliorates cardiac fibrosis and downregulates BAX, type I collagen, interleukin 6 (IL-6), and tumor necrosis factor alpha (TNF-α) by blocking CD47 signaling. *In vitro* experiments further confirm that aCD47 suppresses phosphorylation of p38 mitogen-activated protein kinase (p38 MAPK). These findings implicate CD47 as a potential therapeutic target in DCM (Hao et al., 2022).

#### 2.5.4 Metabolic dysregulation axis

DOX initially suppresses the peroxisome proliferator-activated receptor alpha (PPARα)/PPAR gamma coactivator 1 alpha (PGC-1α) signaling pathway, precipitating mitochondrial dysfunction, metabolic derangement, and excessive ROS accumulation. The combined effects of ROS toxicity and disrupted energy homeostasis subsequently activate the apoptotic program, characterized by Bax upregulation, Bcl-2 suppression, cytochrome c release, and initiation of the caspase dependent cascade (Yang et al., 2015).

#### 2.5.5 Molecular chaperone regulation

Heat shock protein 27 (Hsp27), also known as heat shock protein family B small member 1 (HSPB1), is a core member of the small heat shock protein family and plays an essential role in cellular stress responses and homeostasis maintenance (Shan et al., 2021). Overexpression of Hsp27 improves survival and cardiac function in DOX-treated mice. *In vitro*, Hsp27 overexpression suppresses p53 transcriptional activity and reduces nuclear p53 abundance, an effect that is abolished in mutant Hsp27. Only wild-type Hsp27 overexpression lowers Bax levels, elevates phosphorylated mTOR, and diminishes poly (ADP-ribose) polymerase 1 (PARP-1) cleavage. The p53 inhibitor pifithrin-α (PFT-α) enhances the survival of cardiomyocytes lacking Hsp27 overexpression or expressing mutant Hsp27. Co-immunoprecipitation (CO-IP) reveals that only wild-type Hsp27 binds p53 efficiently; phosphorylation-defective mutants exhibit markedly reduced p53 binding. Thus, phosphorylation of Hsp27 is requisite for p53 interaction, downstream apoptotic regulation, and cardioprotection (Kanagasabai et al., 2021).

#### 2.5.6 Cytoskeleton-associated signaling

Cypher/ZASP, encoded by the LIM domain binding 3 (LDB3) gene, is a PDZ-LIM protein predominantly expressed in cardiac and skeletal muscle. Its PDZ domain mediates binding to α-actinin-2 and localization to the Z-line, thereby preserving sarcomeric integrity (Lopez-Ayala et al., 2015; Peter et al., 2011). LDB3 deficiency is strongly linked to human cardiomyopathy (Zheng et al., 2010). Cypher knockdown promotes cardiomyocyte apoptosis both *in vivo* and *in vitro*. In H9C2 cells treated with Cypher siRNA, p38 MAPK phosphorylation increases, ERK1/2 phosphorylation decreases, and JNK activity remains unchanged. Co-immunoprecipitation reveals Cypher colocalization with AKT, and the AKT activator SC79 reverses the upregulation of pro-apoptotic proteins induced by Cypher loss. These data establish the AKT/p38 MAPK axis as a critical regulator of apoptosis triggered by Cypher deficiency (Xuan et al., 2020).

#### 2.5.7 Cell cycle regulation

The E2F transcription factor/retinoblastoma protein (E2F/Rb) pathway constitutes a central signaling network governing cell cycle progression, differentiation, and apoptosis; its dysregulation is implicated in diverse pathological states (Korenjak and Brehm, 2005). E2F transcription factor 6 (E2F6), a key repressor within this pathway, suppresses apoptosis in neonatal cardiomyocytes under cobalt chloride (CoCl_2_) stress. Overexpression of E2F6, however, fails to confer protection against DOX. Mechanistically, DOX inhibits alpha myosin heavy chain (α-MHC) promoter activity, thereby reducing E2F6 transcription, while simultaneously promoting proteasomal degradation of the E2F6 protein (marked decline at 6 h, near complete loss by 12 h) (Major et al., 2017). This regulation exhibits cell type specificity: in HeLa cells, DOX upregulates E2F transcription factor 1 (E2F1), which activates checkpoint kinase 1 (Chk1) to target E2F6 for proteasomal destruction, thereby driving apoptosis (Major et al., 2017). These divergent responses underscore the context-dependent functional complexity of the E2F/Rb pathway.

Apoptosis in DOX-induced DCM arises from multilayered crosstalk involving metabolic dysregulation, mitochondrial injury, inflammatory activation, and cell cycle disruption. The pathways delineated above, however, have largely been validated in isolation using single-model systems. Their hierarchical relationships, synergistic interactions, and relative quantitative contributions to DOX cardiotoxicity remain poorly integrated.

### 2.6 Apoptosis in myocarditis-associated DCM

Myocarditis represents a critical precursor to DCM, with disease progression driven by sustained inflammation, cell death, and oxidative stress (Mason, 2003). Lipoxin derivatives serve as endogenous pro-resolution mediators (Zhou and You, 2022). The synthetic lipoxin A4 analog BML-111 exerts protective effects in experimental autoimmune myocarditis (EAM) mice by suppressing cardiomyocyte apoptosis, as evidenced by reduced terminal deoxynucleotidyl transferase dUTP nick end labeling (TUNEL) positive cells, diminished cleaved caspase-3 and endonuclease G (EndoG) expression, and preserved Bcl-2 levels (Godson et al., 2023). Mechanistically, BML-111 antagonizes Kelch-like ECH-associated protein 1 (Keap1)-mediated inhibition of nuclear factor erythroid 2-related factor 2 (Nrf2), promoting Nrf2 nuclear translocation and subsequent transcription of antioxidant genes. This process depends on upstream activation of calcium/calmodulin-dependent protein kinase 2 (CaMKK2), which phosphorylates AMPKα, thereby establishing the CaMKK2–AMPKα–Nrf2 signaling axis (Jaén et al., 2020). Clinical evidence confirming comparable protective efficacy of this axis in human myocarditis-related DCM remains lacking. In coxsackievirus B3 (CVB3)-induced DCM mice, IL-6 knockout suppresses cardiomyocyte apoptosis, improves cardiac function, and attenuates fibrosis, effects mechanistically linked to impaired signal transducer and activator of transcription 3 (STAT3) phosphorylation (Li et al., 2019). Whether the pro-resolving BML-111 axis and the pro-inflammatory IL-6/STAT3 axis converge on shared downstream effectors, such as Nrf2 or the Bcl-2 family, has yet to be investigated.

Collectively, apoptosis in the context of myocardial inflammation is governed by bidirectional inputs from pro-resolving and pro-inflammatory signals. The functional hierarchy and points of crosstalk between these pathways remain to be integrated.

### 2.7 Additional molecular mechanisms linked to apoptosis

Sirtuin 1 (SIRT1), a nicotinamide adenine dinucleotide (NAD^+^) dependent deacetylase, exerts protective effects in cardiovascular disease (Chen et al., 2020; D’onofrio et al., 2018). In mice with cardiac-specific expression of a dominant-negative SIRT1 mutant (SIRT1^H363Y^), suppressed SIRT1 activity elevates p53 acetylation and upregulates Bax, thereby triggering cardiomyocyte apoptosis and accelerating DCM progression and mortality (Mu et al., 2014). Systematic characterization of endogenous SIRT1 expression dynamics and its upstream regulatory signals in DCM, however, remains lacking.

The Hippo pathway is an evolutionarily conserved signaling cascade that governs organ size, cell proliferation, and apoptosis (Ma et al., 2019). In mammals, STE20-like kinase 1 (Mst1) serves as a core kinase of this pathway. Glyceraldehyde-3-phosphate dehydrogenase (GAPDH) directly binds Mst1 and modulates its activity, thereby promoting cardiomyocyte apoptosis and contributing to DCM pathogenesis (You et al., 2013). This finding establishes a direct link between the metabolic enzyme GAPDH and Hippo signaling. Whether the GAPDH–Mst1 interaction is regulated by metabolic status, such as glycolytic flux or NAD^+^ levels, remains unclear.

Apoptosis represents a central mechanism underlying cardiomyocyte loss in DCM. Its execution hinges on the caspase family cascade, orchestrated jointly by the extrinsic death receptor pathway and the intrinsic mitochondrial pathway, and is tightly governed by the Bcl-2 protein family. In DCM, diverse pathological insults converge upon this apoptotic signaling network. Genetically, TTNtvs initiate apoptosis *via* the NF-κB-IER3 axis. Epigenetically, non-coding RNAs (including miR-185-5p, miR-186-5p, circ23679, LINC00339, and lncRNA H19) and aberrant DNA methylation, exemplified by chromatin reorganization and CpG methylation alterations in LMNA-related DCM, collectively remodel apoptotic gene expression. Persistent ER stress drives apoptosis through the PERK/ATF6/IRE1–CHOP axis. CYP2E1 overexpression exacerbates oxidative stress and mitochondrial damage, whereas CYP2J2 exerts functional antagonism by inducing protective autophagy *via* AMPK/mTOR. DOX cardiotoxicity engages multilayered mechanisms, including metabolic derangement, mitochondrial dysfunction, and E2F6 degradation. In the context of myocardial inflammation, the BML-111-activated CaMKK2–AMPKα–Nrf2 axis and the IL-6/STAT3 axis confer anti-apoptotic and pro-apoptotic effects, respectively. SIRT1 and the Hippo pathway also participate in apoptotic regulation, though their functional interplay remains poorly defined.

**FIGURE 1:**
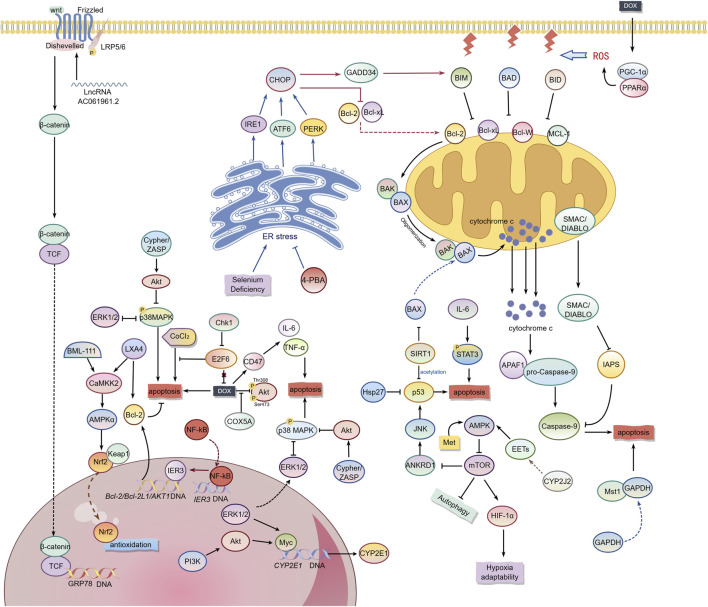
Core apoptotic signaling pathways and regulatory mechanisms in DCM. Core Execution Pathway of Apoptosis. Under stress, pro-apoptotic members of the Bcl-2 family, including BIM, BID, and BAD, are activated and subsequently inhibit anti-apoptotic proteins such as Bcl-2, BCL-xL, BCL-W, and MCL-1. This inhibition permits activation of BAX and BAK, which oligomerize on the mitochondrial outer membrane to induce MOMP. MOMP facilitates the release of cytochrome c and SMAC/DIABLO into the cytosol. Cytochrome c assembles with APAF1 and pro-caspase-9 to form the apoptosome, thereby activating caspase-9. Active caspase-9 then cleaves and activates the executioner caspases, caspase-3 and caspase-7. Simultaneously, SMAC/DIABLO antagonizes IAPs to further potentiate caspase activation. Persistent ER stress activates the UPR through sensors including PERK, IRE1, and ATF6, leading to upregulation of the transcription factor CHOP. CHOP modulates apoptosis-related genes, including Bcl-2, BIM, and GADD34, thereby promoting apoptosis. Regulatory network of apoptosis in DCM. (1) Genetic regulation. NF-κB undergoes nuclear translocation, binds the IER3 promoter, and drives its transcription. The translated IER3 protein shuttles into the nucleus, where it engages promoter regions of apoptotic genes to upregulate protective factors, including Bcl-2, Bcl-2L1, and AKT1. (2) Endoplasmic reticulum stress pathway. Sustained ER stress activates the UPR via PERK, IRE1, and ATF6, upregulating CHOP and GADD34. This shifts the balance of Bcl-2 family proteins, including Bcl-2, Bcl-xL, and BIM, toward apoptosis. lncRNA AC061961.2 activates WNT/β-catenin signaling, promoting binding of the β-catenin/TCF complex to the GRP78 promoter and enhancing its transcription, which improves protein folding and suppresses ER stress and cardiomyocyte apoptosis. Selenium deficiency evokes ER stress and oxidative stress; the chemical chaperone 4-PBA alleviates selenium deficiency-induced cardiomyocyte apoptosis by inhibiting ER stress signaling. (3) Autophagy and metabolic stress. CYP2E1 overexpression exacerbates oxidative stress and promotes apoptosis. Its transcription is driven by Myc, which is itself subject to coordinated regulation by ERK1/2 and PI3K/AKT signaling. EETs generated by CYP2J2 induce protective autophagy via the AMPK/mTOR pathway. mTOR also suppresses apoptosis through the ANKRD1/JNK/p53 axis. (4) DOX-induced apoptosis. DOX inhibits the PPARα/PGC-1α pathway, causing mitochondrial dysfunction and ROS accumulation, which trigger apoptosis. COX5A downregulation reduces SOD2 levels, alleviating oxidative stress and counteracting DOX-mediated suppression of AKT phosphorylation. CD47 upregulates IL-6 and TNF-α, promoting apoptosis. Hsp27 binds and inhibits the transcriptional activity of p53. The cytoskeletal protein Cypher/ZASP regulates apoptosis via the AKT/p38 MAPK axis. E2F6 suppresses apoptosis in NCMs under CoCl_2_ stress, whereas DOX activates Chk1 to markedly reduce E2F6 transcription and target the E2F6 protein for proteasomal degradation. (5) Inflammation and oxidative Stress. IL-6 promotes apoptosis through STAT3 signaling. The lipoxin analog BML-111 suppresses oxidative stress and apoptosis *via* the CaMKK2–AMPKα–Nrf2 axis. (6) Additional key regulatory nodes. SIRT1 regulates the pro-apoptotic activity of p53 through deacetylation. GAPDH directly binds the Hippo pathway kinase Mst1, promoting apoptosis.

**FIGURE 2:**
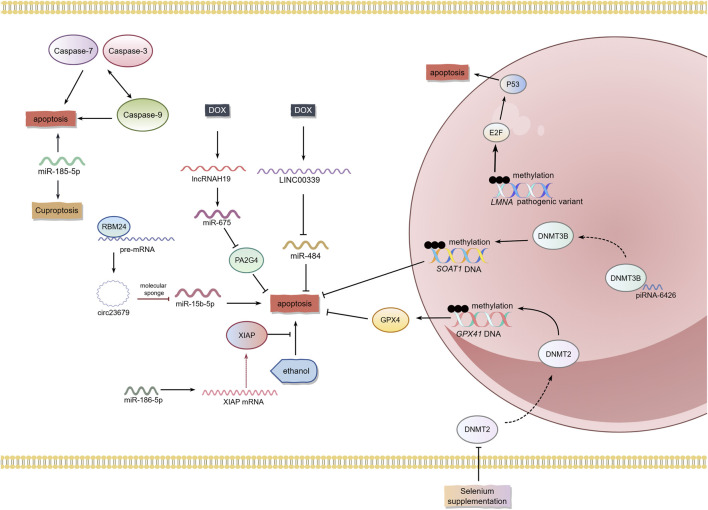
Core apoptotic signaling pathways and epigenetic regulatory mechanisms in DCM. (1) miR-185-5p promotes both apoptosis and cuproptosis. (2) RBM24 directly binds pre-mRNA to generate circ23679, which functions as a molecular sponge for miR-15b-5p, thereby preventing miR-15b-5p-induced apoptosis. (3) DOX upregulates LINC00339 expression, enhancing the suppression of miR-484 and promoting apoptosis. (4) DOX induces upregulation of lncRNA H19 and miR-675, which target PA2G4 to facilitate apoptosis. (5) miR-186-5p directly targets the XIAP mRNA, upregulating XIAP expression and inhibiting ethanol-induced cardiomyocyte apoptosis. (6) Selenium supplementation inhibits DNMT2-mediated promoter methylation of GPX1, upregulates GPX1 expression, and suppresses cardiomyocyte apoptosis. (7) LMNA pathogenic variants directly induce cardiomyocyte apoptosis through the E2F/P53 pathway. (8) piRNA-6426 promotes DNMT3B enrichment at the SOAT1 promoter, modulates its methylation, and inhibits cardiomyocyte apoptosis.

These findings collectively delineate a multidimensional regulatory network governing apoptosis in DCM. Most pathways, however, have been validated only in isolated model systems. Their relative contributions, hierarchical relationships, and synergistic or antagonistic interactions across distinct etiological subtypes and disease stages in human DCM remain poorly integrated. Elucidating the nodal regulatory logic of this network will establish a more precise molecular foundation for developing targeted interventions. Table 2 summarizes the intervention targets and experimental models of apoptosis. Figure 1 depicts the core signaling pathways of apoptosis and their regulatory mechanisms in dilated cardiomyopathy, while Figure 2 illustrates the associated epigenetic regulatory mechanisms.

**TABLE 2:** Apoptosis intervention targets and experimental models.

Apoptosis target	Therapeutic strategy or agent	Brief description of core mechanism	Experimental model
Genetic/epigenetic regulation			
IER3 (Zhou et al., 2017)	Upregulation of IER3 expression	Promotes NF-κB nuclear translocation and upregulates Bcl-2, Bcl-2L1, and AKT1 to suppress apoptosis	TTN truncation heterozygous DCM mouse model
miR-185-5p (Xu et al., 2025)	Cardiac-targeted liposomal miR-185-5p inhibitor	Reduces pro-apoptotic BAX and cleaved caspase-3 and increases anti-apoptotic Bcl-2	*In vitro* cell assays plus *in vivo* mouse studies (cardiac-targeted liposomal delivery)
circ23679 (Liu et al., 2025)	Overexpression of circ23679	Acts as a molecular sponge for miR-15b-5p, relieving target gene repression and inhibiting cardiomyocyte apoptosis	Heart failure mouse model (circ23679 overexpression)
LINC00339 (Li et al., 2018)	Knockdown of LINC00339	Relieves negative regulation of miR-484, promotes proliferation, and inhibits apoptosis	DOX-treated primary cardiomyocytes
lncRNA H19/miR-675 (Zhang et al., 2017)	Targeting the lncRNA H19/miR-675 axis	Suppresses miR-675-mediated negative regulation of PA2G4, thereby attenuating apoptosis	DOX-induced DCM model; miR-675 mimic-transfected cardiomyocytes
miR-186-5p (Liu and Yu, 2019)	Inhibition of miR-186-5p	Upregulates endogenous XIAP to antagonize ethanol-induced apoptosis	Ethanol-treated AC16 human cardiomyocyte cell line
HDAC6 (Yang et al., 2022)	HDAC6 inhibitor TYA-018	Alleviates sarcomeric injury, improves mitochondrial function, and reduces cardiomyocyte apoptosis	BAG3-knockout DCM mouse model
HDAC5 (Hasan et al., 2025)	HDAC5 knockdown; class IIa HDAC inhibitor TMP-195	Reverses cardiac dysfunction and profibrotic gene expression induced by TTN deficiency	TTNtv-associated DCM model
HDACs(I/II类) (Mikšiūnas et al., 2024)	Class I/II HDAC inhibitors	Improve acetylation status in DCM myocardial stem cells, activate cardiac transcription factors, and upregulate structural proteins	Human myocardial mesenchymal stem cells derived from DCM patients
Endoplasmic reticulum stress-related			
LncRNA AC061961.2 (Qiu et al., 2021)	Upregulation of lncRNA AC061961.2	Activates WNT/β-catenin signaling, enhances GRP78 transcription, improves protein folding, and inhibits ER stress	DCM patient myocardial tissue (validation of downregulation); *in vitro* overexpression systems
ER stress pathway (Shan et al., 2015)	Chemical chaperone 4-phenylbutyrate (4-PBA)	Inhibits ER stress signaling and alleviates selenium deficiency-induced cardiomyocyte apoptosis	Selenium-deficient rat model
Autophagy/metabolic regulation			
CYP2E1 (Lu et al., 2012; Pang et al., 2021)	Knockdown of CYP2E1 or inhibitor diallyl sulfide (DAS)	Suppresses oxidative stress and mitochondria-dependent apoptosis	cTnT^R141W^ transgenic DCM mouse model
CYP2J2/EETs (Guan et al., 2019)	Upregulation of CYP2J2 or supplementation with EETs	Induces protective autophagy *via* AMPK/mTOR and reduces apoptosis	Ethanol-induced cardiac dysfunction model
AMPK/mTOR axis (Zhou et al., 2018)	Metformin	Activates AMPK, inhibits mTOR, enhances autophagy, and attenuates oxidative stress and apoptosis	DOX-treated mouse heart tissue
DOX-induced DCM specific			
COX5A (Zhang P et al., 2023)	Overexpression of COX5A	Restores AKT phosphorylation, improves mitochondrial function, and inhibits oxidative stress and caspase activation	DOX-treated mouse heart tissue and H9C2 cardiomyocytes
CD47 (Hao et al., 2022)	CD47 neutralizing antibody (aCD47)	Inhibits p38 MAPK phosphorylation and downregulates BAX, collagen I, IL-6, and TNF-α	DOX-induced DCM mouse model; *in vitro* assays
p53 (Kanagasabai et al., 2021)	Hsp27 overexpression; p53 inhibitor PFT-α	Hsp27 binds and inhibits p53 transcriptional activity, lowering Bax and restoring mTOR activity; PFT-α improves cell survival	DOX-treated mice (*in vivo*); Hsp27-overexpressing cardiomyocytes (*in vitro*)
Cypher/ZASP (Xuan et al., 2020)	AKT activator SC79	Reverses AKT downregulation and p38 MAPK pro-apoptotic signaling caused by Cypher loss	Cypher siRNA-treated H9C2 cells; *in vitro* and *in vivo* Cypher knockdown models
E2F6 (Major et al., 2017)	Stabilization of E2F6 protein (inhibition of degradation)	DOX induces E2F6 degradation; maintaining E2F6 stability suppresses cardiomyocyte apoptosis	DOX-treated neonatal cardiomyocytes (NCMs); HeLa cells (cell-type-dependent validation)
Inflammation/immune-related			
Nrf2 pathway (Jaén et al., 2020)	Lipoxin A4 analog BML-111	Promotes Nrf2 nuclear translocation *via* the CaMKK2-AMPKα axis, inhibiting apoptosis and oxidative stress	Experimental autoimmune myocarditis (EAM) mouse model
IL-6/STAT3 (Li et al., 2019)	IL-6 knockout or inhibition of STAT3 phosphorylation	Suppresses inflammation-associated apoptosis and improves cardiac function and fibrosis	CVB3-induced DCM mouse model (IL-6 knockout)
Other core pathways			
SIRT1 (Mu et al., 2014)	Enhancement of SIRT1 deacetylase activity	Reduces p53 acetylation and downregulates pro-apoptotic Bax expression	Cardiac-specific dominant-negative SIRT1 (SIRT1^H363Y^) transgenic mice
Hippo/Mst1 (You et al., 2013)	Modulation of GAPDH-Mst1 interaction	Inhibition of Mst1 kinase activity attenuates cardiomyocyte apoptosis	Mammalian cell/animal models (validation of GAPDH-Mst1 binding)

## 3 Necroptosis in DCM

Necroptosis is a programmed form of necrosis engaged when apoptosis is compromised or phagocytic clearance fails. Characterized by cellular swelling, membrane lysis, and release of pro-inflammatory mediators, it constitutes an inflammatory PCD modality (Dondelinger et al., 2016; Kearney and Martin, 2017; Ucker, 2016). Signal initiation depends on activation of death receptors (tumor necrosis factor receptor, TNFR; Fas) or toll-like receptors (TLR3 and TLR4). Adaptor proteins, including tumor necrosis factor receptor type 1-associated death domain protein (TRADD), Fas-associated death domain (FADD) protein, and TIR domain-containing adapter-inducing interferon-β (TRIF), recruit receptor-interacting serine/threonine kinase 1 (RIPK1) together with caspase-8 or caspase-10 (Zhou et al., 2024; Guo et al., 2022). At steady state, RIPK1 is maintained in an inactive state through ubiquitination mediated by IAPs. Upon receipt of a death stimulus, the deubiquitinase CYLD removes these ubiquitin moieties, liberating RIPK1 to recruit receptor-interacting serine/threonine kinase 3 (RIPK3) and form a heteromeric complex (Ye et al., 2023). RIPK3 subsequently phosphorylates mixed lineage kinase domain-like pseudokinase (MLKL). Phosphorylated MLKL undergoes inositol hexaphosphate (IP6)-driven oligomerization into a necrosome, which translocates to phosphatidylinositol phosphate (PIP)-enriched microdomains of the plasma membrane and assembles transmembrane pores. The resultant influx of ions precipitates cellular swelling and extrusion of intracellular contents (Petrie et al., 2019).

Although the core signaling architecture of necroptosis is reasonably well defined, evidence for its activation in DCM cardiomyocytes derives largely from indirect indices, such as altered expression of RIPK1, RIPK3, or MLKL. Direct functional validation, specifically whether MLKL pore formation constitutes a critical execution step in DCM-associated cardiomyocyte loss, remains sparse. Furthermore, while EAM models have demonstrated that apoptotic blockade can provoke compensatory activation of necroptosis (Wu Y. et al., 2021), the precise transition nodes and relative contributions of these two death modalities across distinct etiological subtypes and disease stages of DCM have yet to be systematically evaluated.

### 3.1 Validation and activation of necroptosis in DCM

Analysis of myocardial biopsies from 56 patients with DCM revealed marked upregulation of phosphorylated MLKL (p-MLKL) in cardiomyocyte nuclei, cytosol, and intercalated discs, with particularly pronounced nuclear signals in hypertrophic cardiomyocytes (Fujita et al., 2022). Nuclear p-MLKL levels correlated negatively with left ventricular diastolic function indices and positively with right heart loading parameters, whereas intercalated disc p-MLKL correlated negatively with mean left ventricular wall thickness. These distinct associations imply that subcellular localization confers specific functional significance (Fujita et al., 2022). Prognostically, patients with high nuclear p-MLKL exhibited a 32% adverse event rate, compared with 4% in the low nuclear p-MLKL group; no such association was observed for intercalated disc p-MLKL. Nuclear p-MLKL accumulation, thus, represents a key feature linked to progressive ventricular dysfunction and poor outcomes in DCM. These findings were recapitulated in Sgcd-deficient mice (Fujita et al., 2022). Additionally, DCM myocardium displays a selective increase in MLKL phosphorylated at Thr^357^ (pThr^357^-MLKL), with no significant change at Ser^358^, implicating pThr^357^ as a potential core site for pathological necroptotic activation (Szobi et al., 2017).

Although these clinical data establish a robust association between nuclear p-MLKL localization and adverse DCM prognosis, the evidence remains correlative. Whether nuclear p-MLKL directly participates in transcriptional regulation or chromatin remodeling to drive cardiomyocyte dysfunction awaits functional validation through experimental studies. Whether the selective elevation of pThr^357^-MLKL varies across DCM etiological subtypes, including genetic, inflammatory, or drug-induced forms, also remains undefined.

### 3.2 Necroptosis in desmoplakin cardiomyopathy

Desmoplakin, encoded by the desmoplakin (*DSP*) gene, is a core constituent of intercalated disc desmosomes. Together with β-catenin in adherens junctions, it maintains the structural integrity of the cardiac intercalated disc (Chopra et al., 2011; Buckley et al., 2014). Pathogenic variants in DSP give rise to a continuous phenotypic spectrum encompassing arrhythmogenic cardiomyopathy and DCM (Hatsell and Cowin, 2001; Norman et al., 2005; Gigli et al., 2019). Loss of DSP disrupts desmosomal architecture and compromises intercalated disc structure, leading to aberrant β-catenin localization. Because β-catenin also functions as a transcriptional co-activator in the canonical WNT (cWNT) pathway, its mislocalization may further perturb downstream signal transduction (Chopra et al., 2011; Buckley et al., 2014).

In mice with cardiomyocyte-specific Dsp deletion induced at 2 weeks of age (Myh6-McmTam:Dsp^F/F^)cWNT/β-catenin signaling is profoundly dysregulated, manifesting as increased transcription of downstream targets, elevated expression of co-effector isoforms, and concurrent upregulation of endogenous pathway inhibitors. Concomitantly, myocardial levels of necroptotic and apoptotic markers, including RIPK1, RIPK3, MLKL, and cleaved caspase-3, increase substantially. Functional rescue experiments demonstrate that β-catenin inactivation prolongs survival, improves cardiac function, and attenuates fibrosis and cell death. Conversely, β-catenin activation exacerbates disease severity, and these effects occur independently of cWNT transcriptional activity (Olcum et al., 2023a). These observations suggest that inhibiting, rather than activating, β-catenin may represent a potential therapeutic strategy for this cardiomyopathy.

Several critical issues, however, remain unresolved. Whether necroptotic and apoptotic activation in this setting reflects a direct consequence of DSP loss or a secondary event stemming from β-catenin dysregulation is unclear. The molecular link connecting RIPK1, RIPK3, and MLKL to β-catenin has not been defined. Moreover, although Olcum et al. (2023b) identified PANoptosis, an integrated form of cell death encompassing apoptosis, necroptosis, and pyroptosis, as a prominent feature of DSP cardiomyopathy and demonstrated that β-catenin inactivation mitigates PANoptosis (Olcum et al., 2023a), the relative contributions, temporal sequence, and precise inflammasome-related mechanisms of these three death modalities in this model await further elucidation.

### 3.3 Upstream triggers and regulatory pathways of necroptosis

Peroxisome PGC-1α serves as a master regulator of mitochondrial biogenesis and function, with additional roles in antioxidant defense, energy metabolism, and inflammatory or immune modulation (Satish et al., 2018; Dinulovic et al., 2016). In DOX-induced DCM mice, a PGC-1α agonist attenuates oxidative stress, as reflected by an increased glutathione-to-glutathione disulfide (GSH/GSSG) ratio and reduced 8-hydroxy-2′-deoxyguanosine (8-OHdG) levels, and downregulates the necroptotic markers RIPK1, RIPK3, and MLKL. These observations implicate PGC-1α activation as a potential therapeutic avenue (Shipra et al., 2023). Whether PGC-1α modulates necroptosis by directly targeting the RIPK1/RIPK3/MLKL axis or indirectly through preservation of mitochondrial function remains undefined.

*TAB2* encodes TGF-beta activated kinase 1 binding protein 2, a protein involved in immune regulation and adverse ventricular remodeling. Polymorphisms in TAB2 are linked to DCM susceptibility and prognosis (Vasilescu et al., 2018; Shen et al., 2020). Cardiomyocyte-specific *TAB2* knockout mice develop DCM accompanied by extensive apoptosis and necroptosis. Mechanistically, TAB2 promotes transforming growth factor-beta-activated kinase 1 (TAK1)-dependent phosphorylation of RIPK1 at Ser321. This phosphorylation restrains RIPK1 kinase activity, thereby preventing assembly of both the RIPK1–FADD–caspase-8 apoptotic complex and the RIPK1–RIPK3 necroptotic complex. Inactivation of RIPK1 reverses myocardial remodeling and cardiac dysfunction in TAB2-deficient mice (Yin et al., 2022). These findings establish TAB2, acting *via* TAK1-mediated RIPK1 Ser321 phosphorylation, as a critical protective signal that suppresses both cardiomyocyte apoptosis and necroptosis. Notably, although both PGC-1α and TAB2 participate in regulating cardiomyocyte death in DCM, they operate at distinct levels: PGC-1α governs mitochondrial homeostasis, while TAB2 directly modulates RIPK1 kinase activity. Whether these two pathways engage in synergistic or antagonistic crosstalk in DCM, and their relative contributions across distinct DCM etiologies, represent critical gaps in current knowledge.

### 3.4 Interplay among necroptosis, apoptosis, and autophagy

In a porcine cardiac myosin-induced EAM rat model, pharmacological inhibition of necroptosis with Nec-1, autophagy with 3-MA, or apoptosis with the pan-caspase inhibitor zVAD-fmk revealed compensatory crosstalk among these pathways. Blockade of necroptosis redirected cell death toward apoptosis. Inhibition of autophagy triggered a feedback activation of necroptosis. Suppression of apoptosis concurrently enhanced both necroptotic and autophagic activity (Wu Y. et al., 2021). These findings indicate that stressed cardiomyocytes execute death programs through reciprocal compensation among distinct death modalities. Inhibiting any single pathway independently reduced myocardial inflammation and improved cardiac function. Whether these compensatory relationships extend to DOX-induced, genetic, or other DCM subtypes awaits further validation.

Analysis of myocardial tissue from 22 patients undergoing heart transplantation for ischemic cardiomyopathy or DCM revealed widespread autophagy, characterized by intracellular vacuolization and nuclear vacuolar disintegration. TUNEL staining and immunohistochemistry for complement C3, complement C9, RIPK1, and RIPK3 were all positive. Progression of autophagy triggers secondary cell death encompassing both passive oncosis and active necroptosis, each occurring more frequently than apoptosis. In surviving cells, activation of RIPK1/NF-κB signaling suggests that this pathway may exert a compensatory pro-survival role (Corsetti et al., 2019).

Although the EAM model has established compensatory crosstalk among the three PCD modalities and human DCM samples confirm the coexistence and relatively higher frequency of necroptosis and autophagy, the dominant roles, transition triggers, and quantitative contributions of these pathways to cardiomyocyte loss across distinct DCM stages remain poorly defined. Moreover, RIPK1/NF-κB signaling exerts dual pro-death and pro-survival functions in DCM. The contextual determinants governing its net effect represent another critical unresolved question.

Necroptosis represents a prominent inflammatory PCD modality in DCM. Its core signaling axis, comprising RIPK1, RIPK3, and MLKL, is aberrantly activated in the myocardium of patients with DCM and across diverse animal models. Clinical evidence reveals marked upregulation of p-MLKL in cardiomyocyte nuclei, cytosol, and intercalated discs. Nuclear p-MLKL accumulation correlates strongly with progressive ventricular dysfunction and adverse outcomes, underscoring its potential biomarker utility (Fujita et al., 2022). Selective elevation of pThr^357^-MLKL further implicates phosphorylation at this residue as a central step in pathological necroptotic activation (Szobi et al., 2017). Mechanistically, DSP deficiency in desmoplakin cardiomyopathy drives PANoptosis activation through β-catenin dysregulation (Olcum et al., 2023a; Olcum et al., 2023b). In DOX-induced DCM, PGC-1α indirectly suppresses necroptosis by preserving mitochondrial function. TAB2, acting *via* TAK1-mediated RIPK1 Ser321 phosphorylation, directly restrains RIPK1 kinase activity, thereby constituting a critical protective signal that antagonizes both apoptosis and necroptosis (Yin et al., 2022). Moreover, EAM models demonstrate compensatory crosstalk among necroptosis, apoptosis, and autophagy (Wu Y. et al., 2021), and human DCM samples confirm that necroptosis and autophagy occur more frequently than apoptosis (Corsetti et al., 2019).

**FIGURE 3:**
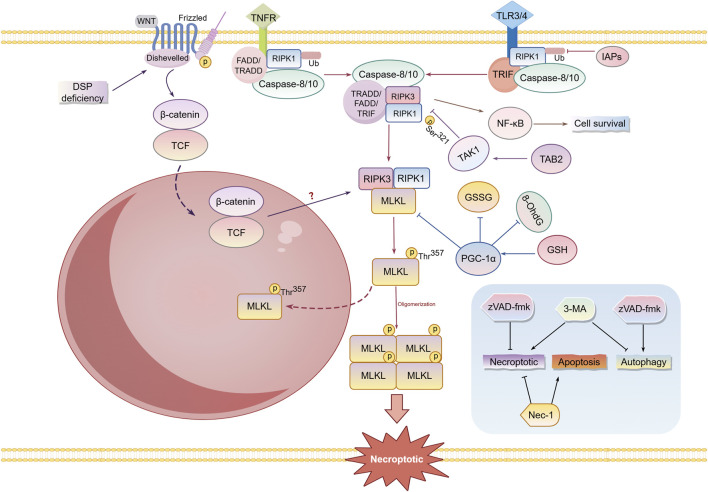
Signaling of necroptosis in dilated cardiomyopathy. Core execution pathway of necroptosis. Necroptosis is triggered by activation of death receptors (TNFR and Fas) or toll-like receptors (TLR3 and TLR4). Receptor engagement recruits adaptor proteins, including FADD, TRADD, or TRIF, which subsequently interact with RIPK1 and caspase-8 or caspase-10. Under basal conditions, IAP-mediated ubiquitination maintains RIPK1 in an inactive state. Deubiquitination of RIPK1 permits recruitment and activation of RIPK3, and together they form the core necrosome complex. This complex phosphorylates MLKL to execute necroptosis. Phosphorylated MLKL can also translocate to the nucleus, further driving cardiac functional deterioration in DCM. Regulatory network of necroptosis in DCM. (1) Activation in specific etiologies. In desmoplakin cardiomyopathy caused by DSP pathogenic variants, loss of DSP aberrantly activates WNT/β-catenin signaling. This process drives upregulation of RIPK1, RIPK3, and MLKL, although the precise underlying mechanism remains undefined. (2) Upstream triggers and negative regulation. PGC-1α, a master regulator of mitochondrial function, increases the GSH/GSSG ratio and reduces RIPK1, RIPK3, and MLKL expression, thereby suppressing necroptosis. TAB2 promotes TAK1-dependent phosphorylation of RIPK1 at Ser321, restraining its kinase activity and consequently blocking assembly of both the RIPK1–FADD–caspase-8 apoptotic complex and the RIPK1–RIPK3 necroptotic complex. (3) Crosstalk with other cell death modalities. Under myocardial stress or inflammation, necroptosis, apoptosis (involving caspase-8), and autophagy exhibit substantial compensatory relationships. Inhibition of any single pathway, such as necroptosis blockade with Nec-1, apoptosis blockade with zVAD-fmk, or autophagy blockade with 3-MA, can redirect cell death toward alternative modalities. Furthermore, excessive autophagy can progress to oncosis or trigger necroptosis, whereas RIPK1 may also activate the NF-κB pathway to promote survival, establishing a compensatory circuit.

Several mechanistic gaps, however, persist. First, whether nuclear p-MLKL directly engages in transcriptional regulation or chromatin remodeling to drive cardiomyocyte dysfunction lacks functional experimental validation. Second, in DSP cardiomyopathy, it remains unclear whether necroptotic and apoptotic activation is a direct consequence of DSP loss or secondary to β-catenin dysregulation; the molecular link connecting RIPK1, RIPK3, and MLKL to β-catenin is also undefined. Third, although PGC-1α and TAB2 regulate necroptosis at distinct levels—mitochondrial homeostasis and RIPK1 kinase activity, respectively—whether these two pathways interact synergistically or antagonistically in DCM, and their relative contributions across distinct DCM etiologies, represent critical knowledge gaps. Fourth, the dominant roles, transition triggers, and quantitative contributions of necroptosis, apoptosis, and autophagy to cardiomyocyte loss across different DCM stages have not been systematically evaluated. Future investigations should prioritize causal validation of these pathways, resolution of their interaction nodes, and quantitative assessment of their stage-specific contributions. Such efforts will establish a more rigorous foundation for developing precision therapeutic strategies targeting necroptosis in DCM. Table 3 summarizes the intervention targets and experimental models of necroptosis. Figure 3 depicts necroptosis signaling in dilated cardiomyopathy.

**TABLE 3:** Necroptosis intervention targets and experimental models.

Necroptosis intervention target	Therapeutic strategy or drug	Brief description of core mechanism	Experimental model
β-catenin (Olcum et al., 2023a)	Inhibition of β-catenin function (non-transcriptional activity)	DSP loss disrupts β-catenin signaling; inhibiting β-catenin mitigates necroptosis and apoptosis, improving myocardial remodeling	Cardiomyocyte-specific Dsp conditional knockout mice (Myh6-McmTam:Dsp^F/F^)
PGC-1α (Shipra et al., 2023)	PGC-1α agonist	Attenuates oxidative stress and downregulates RIPK1, RIPK3, and MLKL expression	DOX-induced DCM mouse model
RIPK1 (Yin et al., 2022)	RIPK1 kinase inhibitor (e.g., Nec-1); genetic inactivation of RIPK1	Blocks RIPK1–RIPK3 necrosome formation; reverses myocardial remodeling and cardiac dysfunction caused by TAB2 deficiency	Cardiomyocyte-specific Tab2 knockout mice
PI3K (Wu Y. et al., 2021)	Autophagy inhibitor 3-MA	Autophagy inhibition independently reduces myocardial inflammation and improves cardiac function but feedback activates necroptosis	Porcine cardiac myosin-induced EAM rat model
Caspase (Wu Y. et al., 2021)	Pan-caspase inhibitor zVAD-fmk	Apoptosis inhibition independently reduces myocardial inflammation and improves cardiac function but enhances necroptosis and autophagy	Porcine cardiac myosin-induced EAM rat model
RIPK1 (Wu Y. et al., 2021)	RIPK1 inhibitor Nec-1	Necroptosis inhibition independently reduces myocardial inflammation and improves cardiac function but redirects cell death toward apoptosis	Porcine cardiac myosin-induced EAM rat model

## 4 Pyroptosis in DCM

Pyroptosis is a form of PCD triggered by inflammasome activation and plays a central role in immune responses and inflammatory processes (Yu et al., 2021). Its hallmark morphological features include plasma membrane pore formation, rapid membrane rupture, cellular swelling and lysis, and the release of intracellular contents, most notably large quantities of pro-inflammatory mediators. These mediators act on neighboring cells and recruit diverse inflammatory cells to the local microenvironment, thereby initiating and amplifying downstream inflammatory cascades that further accelerate pyroptotic cell death (Chai et al., 2022).

Pyroptosis proceeds through two principal routes: a canonical pathway dependent on caspase-1 and a non-canonical pathway dependent on caspase-4, caspase-5, or caspase-11. Both are intimately linked to inflammasome activation. Among the inflammasomes, the NLRP3 inflammasome is the most extensively characterized and representative (Bertheloot et al., 2021; Zhaolin et al., 2019). In the canonical pathway, apoptosis-associated speck-like protein containing a caspase recruitment domain (ASC) recruits and activates caspase-1. Active caspase-1 specifically cleaves gasdermin D (GSDMD), liberating its N-terminal domain, which translocates to the plasma membrane to form pores and thereby initiates pyroptosis. Concurrently, caspase-1 cleaves the precursors pro-interleukin-1β (pro-IL-1β) and pro-interleukin-18 (pro-IL-18) to generate biologically active IL-1β and IL-18, which are released through the GSDMD pores. This amplifies local inflammation and reinforces the pyroptotic process (Yu et al., 2021). Potassium efflux through the membrane pores exerts positive feedback on the NLRP3/ASC/caspase-1 signaling axis, further potentiating inflammasome activation (Chai et al., 2022). In the non-canonical pathway, caspase-3 cleaves gasdermin E (GSDME), releasing its N-terminal fragment to form pores and trigger pyroptosis (Vasudevan et al., 2023).

Although the core signaling framework of pyroptosis is relatively well defined, its investigation in DCM remains in an early stage. Current evidence derives largely from altered expression of NLRP3 inflammasome components and downstream effectors. Direct functional validation that GSDMD- or GSDME-mediated pore formation constitutes a critical execution step in DCM-associated cardiomyocyte loss is lacking. Moreover, the relative contributions of the canonical and non-canonical pathways across distinct etiological subtypes and disease stages of DCM have yet to be systematically assessed.

### 4.1 Validation and activation of cardiomyocyte pyroptosis in DCM

Earlier DCM studies largely relied on TUNEL staining to identify cardiomyocyte apoptosis. DNA fragmentation, however, is not unique to apoptosis; pyroptotic cells also exhibit TUNEL positivity, implying that prior investigations may not have accurately distinguished between the two (Wu et al., 2018).

Zeng et al. (2020) performed the first analysis of left ventricular free-wall myocardium from patients with DCM undergoing cardiac transplantation. Compared with controls, DCM myocardium displayed marked upregulation of the NLRP3 inflammasome, IL-1β, and IL-18, together with pronounced GSDMD cleavage and ASC speck formation, confirming activation of the pyroptotic pathway. Triple immunofluorescence staining for α-actinin, TUNEL, and caspase-1 permitted precise discrimination, revealing a higher proportion of pyroptotic than apoptotic cardiomyocytes in DCM. Mechanistically, NADPH oxidase 1 (NOX1) and NADPH oxidase 4 (NOX4) promote drop-like 1 (Drop1)-mediated mitochondrial fission, exacerbating ROS accumulation and thereby activating the NLRP3 inflammasome to drive pyroptosis. Dual inhibition of NOX1 and NOX4 effectively suppresses cardiomyocyte pyroptosis and alleviates cardiac dysfunction (Zeng et al., 2020). Additional evidence indicates that pyroptosis modulates immune cell infiltration and shapes the myocardial immune microenvironment, playing a pivotal role in DCM immunopathology (Wang K. et al., 2022).

In summary, cardiomyocyte pyroptosis is robustly activated in DCM and may exceed apoptosis in magnitude. These findings, however, derive predominantly from cross-sectional observations of end-stage heart failure explants and validation of a single NOX–ROS–NLRP3 mechanistic axis. The dynamics of pyroptosis during early-stage DCM and across distinct etiological subtypes, direct functional evidence for GSDMD- or GSDME-mediated pore formation, and the relative contributions of canonical versus non-canonical pathways await systematic elucidation. Targeting key pyroptotic effectors holds promise for developing novel DCM therapies.

### 4.2 Regulation of pyroptosis in DCM

Inhibitor of nuclear factor kappa B kinase epsilon (IKKε), a prominent member of the serine/threonine protein kinase family, functions as a central signaling molecule in inflammatory and cellular regulation (Li and Verma, 2002). In a DOX-induced DCM model, IKKε knockout suppresses phosphorylation of inhibitor of NF-κB alpha (IκBα), p65, RelB, and p100 within the NF-κB pathway, thereby attenuating myocardial injury and improving cardiac function (Liu Y. et al., 2022). These observations implicate IKKε as a potential therapeutic target in DCM.

Transient receptor potential ankyrin 1 (TRPA1) is a non-selective cation channel involved in pain perception, inflammation, and cardiovascular regulation (Talavera et al., 2020). Gene Expression Omnibus (GEO) analyses reveal elevated TRPA1 expression in left ventricular tissue from both DCM patients and DCM rats. TRPA1 deficiency exacerbates DOX-induced M1 macrophage polarization, oxidative stress, and cardiomyocyte apoptosis and pyroptosis (Wang M. et al., 2023). RNA sequencing demonstrates that TRPA1 knockout upregulates the inflammatory molecule S100 calcium binding protein A8 (S100A8), which suppresses M1 polarization of bone marrow-derived macrophages. Exogenous recombinant S100A8 potentiates apoptosis, pyroptosis, and oxidative stress in DOX-stimulated primary cardiomyocytes. Activation of TRPA1 with cinnamaldehyde significantly improves cardiac function and downregulates S100A8 expression in DCM rats (Wang M. et al., 2023).

Collectively, TRPA1 loss promotes M1 macrophage polarization and cardiomyocyte apoptosis and pyroptosis through S100A8 upregulation, accelerating DCM progression. It should be noted, however, that both pathways have been validated exclusively in DOX-induced DCM models. Their generalizability to genetic, inflammatory, or other DCM subtypes remains uncertain. Furthermore, whether the IKKε/NF-κB axis and the TRPA1/S100A8 axis functionally interact in regulating pyroptosis and whether a direct causal relationship links NF-κB activation to GSDMD- or GSDME-mediated pore formation have yet to be elucidated.

### 4.3 Regulation of pyroptosis in DCM-related cardiomyopathies

Autoimmune myocarditis, characterized by excessive cardiac inflammation, can progress to DCM and irreversible heart failure (Won et al., 2024). In the EAM mouse model, empagliflozin (EMPA) reduces NF-κB nuclear translocation and inhibits IκBα degradation, thereby blocking the NF-κB pathway. This suppression attenuates downstream pyroptosis, diminishes cardiac inflammation, and improves cardiac function (Won et al., 2024).

Although this study establishes an association between NF-κB inhibition and reduced pyroptosis, whether EMPA directly targets GSDMD- or GSDME-mediated pore formation or acts indirectly through other anti-inflammatory mechanisms remains undefined. Furthermore, the drivers and pathway preferences for pyroptotic activation may differ between autoimmune myocarditis-associated DCM and primary DCM. The generalizability of these findings thus awaits further validation.

**FIGURE 4:**
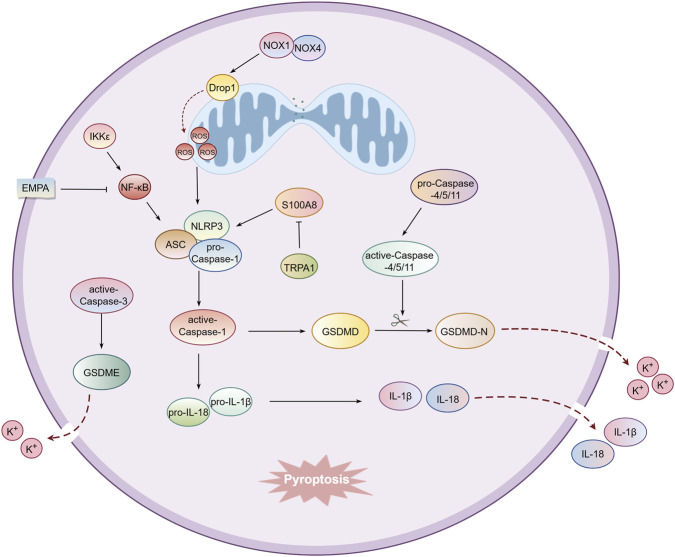
Signaling of pyroptosis in dilated cardiomyopathy. Core execution pathway of pyroptosis. In the canonical pathway, inflammasomes such as NLRP3 are activated. During inflammasome assembly, the adaptor ASC recruits and activates caspase-1. Active caspase-1 cleaves GSDMD, liberating its pore-forming N-terminal domain, and simultaneously processes pro-IL-1β and pro-IL-18 into mature IL-1β and IL-18. These inflammatory mediators are released through the membrane pores, amplifying the inflammatory response. Potassium efflux exerts positive feedback that further reinforces NLRP3/ASC/caspase-1 signaling. The non-canonical pathway is triggered by direct cleavage of GSDMD by active caspase-4, caspase-5, or caspase-11. Additionally, caspase-3 can cleave GSDME, initiating GSDME-dependent pyroptosis. Regulatory network of pyroptosis in DCM. (1) Upstream activation mechanisms. NOX1 and NOX4 promote Drop1-mediated mitochondrial fission, increasing ROS accumulation and thereby activating the NLRP3 inflammasome to drive pyroptosis. (2) Key regulatory nodes. IKKε activates the NF-κB pathway, leading to subsequent NLRP3 inflammasome activation and pyroptosis. TRPA1 suppresses expression of the inflammatory mediator S100A8, thereby inhibiting NLRP3 and attenuating cardiomyocyte pyroptosis. (3) Inhibition of the NF-κB Pathway. Pharmacological agents such as EMPA inhibit NF-κB signaling, blocking downstream pro-inflammatory and pro-pyroptotic signals and reducing myocardial pyroptosis and inflammation.

Cardiomyocyte pyroptosis proceeds via the canonical caspase-1/GSDMD pathway and the non-canonical caspase-3/GSDME pathway, characterized by NLRP3 inflammasome activation and release of IL-1β and IL-18. In DCM myocardium, the NLRP3–ASC–caspase-1–GSDMD axis is robustly activated, and pyroptotic cells outnumber apoptotic cells. Multiple upstream pathways, including NOX1/NOX4-ROS, IKKε/NF-κB, TRPA1/S100A8, and EMPA/NF-κB, converge to regulate pyroptosis. Current evidence, however, derives largely from end-stage samples or DOX/EAM models. Direct functional validation of GSDMD/GSDME pore formation, the relative contributions of canonical versus non-canonical pathways, and the nodes of crosstalk with apoptosis remain critical gaps. Targeting pyroptotic executioners or upstream regulatory kinases offers potential therapeutic strategies for DCM, but these approaches require further validation across distinct disease subtypes and stages. Table 4 summarizes the intervention targets and experimental models of pyroptosis. Figure 4 depicts pyroptosis signaling in dilated cardiomyopathy.

**TABLE 4:** Pyroptosis intervention targets and experimental models.

Pyroptosis intervention target	Therapeutic strategy or drug	Brief description of core mechanism	Experimental model
NOX1/NOX4 (Zeng et al., 2020)	Dual inhibitor of NOX1 and NOX4	Suppresses Drop1-mediated mitochondrial fission, reduces ROS accumulation, and blocks NLRP3 inflammasome activation and downstream pyroptosis	Left ventricular free wall myocardium from DCM heart transplant recipients plus *in vitro* cell assays
IKKε (Liu Y. et al., 2022)	IKKε knockout or inhibition	Inhibits phosphorylation of IκBα, p65, RelB, and p100 in the NF-κB pathway, attenuating myocardial injury and cardiac dysfunction	DOX-induced DCM mouse model (IKKε knockout)
TRPA1 (Wang M. et al., 2023)	TRPA1 activator (e.g., cinnamaldehyde)	Activates TRPA1 to downregulate S100A8 expression, suppressing M1 macrophage polarization, oxidative stress, and cardiomyocyte pyroptosis	DOX-induced DCM rat model plus GEO dataset analysis plus primary cardiomyocytes plus bone marrow-derived macrophages (BMDMs)
NF-κB pathway (Won et al., 2024)	Empagliflozin (EMPA)	Reduces NF-κB nuclear translocation, inhibits IκBα degradation, and blocks the NF-κB pathway and subsequent pyroptosis	Experimental autoimmune myocarditis (EAM) mouse model

## 5 Autophagy in DCM

Autophagy is a catabolic process through which cells degrade their own components to recycle materials, with the breakdown products supplying energy and biosynthetic precursors for cellular metabolism (Xu et al., 2025). Under stress, autophagy is typically induced to preserve intracellular homeostasis. Excessive or dysregulated autophagy, however, can lead to structural disintegration and cell death (Del Re et al., 2019; Qiao et al., 2023). Morphologically, autophagy proceeds through the sequential formation of three distinct membrane structures: the phagophore, the autophagosome, and the autolysosome (Zheng et al., 2023). The overall process comprises four stages: initiation, vesicle elongation, membrane fusion, and cargo degradation (Liu S. et al., 2023; Mizushima and Komatsu, 2011). During initiation, AMPK inhibits mTORC1, relieving mTORC1-mediated suppression of the Unc-51-like autophagy-activating kinase 1 (ULK1) complex and promoting autophagosome nucleation. In the elongation phase, JNK phosphorylates Bcl-2 and Bcl-2 interacting mediator of cell death (BIM), disrupting Beclin1/Bcl-2 and Beclin1/BIM complexes to liberate Beclin1. Free Beclin1 activates vacuolar protein sorting 34 (VPS34), generating phosphatidylinositol 3-phosphate (PI3P) and driving vesicle expansion. Subsequently, the ATG5–ATG12–ATG16L1 ubiquitin-like conjugation system lipidates microtubule-associated protein 1 light chain 3 I (LC3-I) to form LC3-II, which anchors to the autophagosomal membrane and completes autophagosome formation. Finally, autophagosomes fuse with lysosomes to form autolysosomes, wherein the sequestered contents are degraded (Liu S. et al., 2023).

Although the core molecular machinery of autophagy is reasonably well defined, investigations in DCM face several critical challenges. Whether autophagy exerts a protective or pathogenic role in different stages and etiological subtypes of DCM remains unsettled. Monitoring of autophagic flux relies heavily on static markers such as LC3-II, and robust functional assessment tools are lacking. The transition nodes and reciprocal regulatory networks linking autophagy with apoptosis, necroptosis, and pyroptosis have yet to be systematically integrated.

### 5.1 Autophagy activation contributes to cardiomyocyte death in patients with DCM

RNA sequencing of left ventricular tissue from 13 patients with DCM revealed differential expression of 13 autophagy-related genes and 3 phagocytosis-related genes. Among these, altered expression of nuclear receptor-binding protein 2 (NRBP2) and calcium binding and coiled-coil domain 2 (CALCOCO2) correlated closely with cardiac dysfunction and adverse remodeling. Increased numbers of autophagic structures further indicated enhanced autophagic activity (Gil-Cayuela et al., 2019).

Corsetti et al. (2019) analyzed myocardial tissue from 22 patients with ischemic cardiomyopathy or DCM, using 11 individuals who died from non-cardiac causes as controls. LC3-II immunostaining demonstrated markedly elevated autophagic levels in DCM myocardium. Cells with advanced autophagy exhibited widespread nuclear vacuolization, acquired a characteristic “strawberry-like” morphology, and stained positively for complement component C3, TUNEL, complement component C9, receptor-interacting protein 1 (RIP1), and RIP3. Cells with less pronounced vacuolization displayed RIP1 and NF-κB positivity but remained negative for other death markers. These findings indicate that progressive autophagy can trigger secondary cell death, with oncosis and necroptosis occurring more frequently than apoptosis, whereas RIP1/NF-κB activation is associated with cell survival (Corsetti et al., 2019).

Although these studies establish clinical evidence for enhanced autophagic activity and its association with cell death in DCM myocardium, notable limitations persist. First, sample sizes are modest (n = 13 and n = 22), and the observations derive from cross-sectional analyses of end-stage disease, precluding assessment of dynamic autophagic changes during disease progression. Second, evaluation of autophagic activity relies heavily on static LC3-II staining; whether autophagic flux is truly unimpeded and whether autophagosome accumulation stems from increased induction or impaired degradation remains unresolved. Third, the functional relevance of differentially expressed genes such as NRBP2 and CALCOCO2 to the core autophagic machinery remains correlative and lacks causal validation.

### 5.2 Degree of autophagy activation correlates with DCM prognosis

The abundance of autophagic structures in the myocardium of DCM patients substantially exceeds that in healthy controls (Gil-Cayuela et al., 2019). Kanamori et al. (2022) employed electron microscopy to demonstrate that DCM patients who achieve left ventricular reverse remodeling (LVRR) exhibit significantly higher numbers of autophagic vacuoles and autolysosomes, as well as elevated cathepsin D expression, compared with those who do not. These findings suggest that the extent of autophagic activation correlates with a favorable prognosis and may serve as a biomarker for predicting LVRR potential.

Critical caveats, however, must be acknowledged. The evidence derives from small cross-sectional cohorts, and a causal relationship between autophagic activity and LVRR has not been established. Whether enhanced autophagy actively drives LVRR or merely represents an epiphenomenon of compensatory myocardial stress responses remains unresolved. Moreover, whether morphological counts of autophagic vacuoles and autolysosomes faithfully reflect functional autophagic flux awaits further validation.

### 5.3 Moderate autophagy activation ameliorates DCM

In DCM mouse models, both LC3-II expression and autophagosome numbers are markedly increased. Rapamycin further activates autophagy by inhibiting the mechanistic target of rapamycin–eukaryotic translation initiation factor 4E binding protein 1 (mTOR–4EBP1) pathway, thereby improving cardiac function (Jin et al., 2020). These observations suggest that upregulation of autophagy may represent a potential therapeutic strategy for DCM.

Mechanistically, mTOR activation suppresses the autophagic-lysosomal pathway (ALP), leading to the accumulation of dysfunctional organelles and misfolded proteins (Caragnano et al., 2019; Moyzis et al., 2015). This is accompanied by reduced nuclear translocation of transcription factor EB (TFEB), accumulation of branched-chain amino acids (BCAAs), decreased expression of protein phosphatase 2C family member (PP2Cm), and elevated expression of microRNA-22 (miR-22). Downregulation of miR-22 partially reverses these molecular abnormalities (Caragnano et al., 2019). In δ-sarcoglycan-deficient DCM mice, the numbers of autophagic vacuoles and lysosomes are increased and AMPK levels are elevated. Metformin enhances autophagic flux through the AMPK/mTOR pathway and improves cardiac function in this model (Kanamori et al., 2019).

Although these studies establish the cardioprotective potential of autophagy activation in DCM, critical gaps remain. First, the protective effects of rapamycin and metformin converge on mTOR and AMPK signaling, yet whether the benefit stems from enhanced clearance of toxic cargo or from autophagy-independent mechanisms has not been rigorously dissected. Second, whether the miR-22/BCAA/PP2Cm axis intersects with the canonical mTOR/AMPK pathway to regulate autophagy remains undefined. Third, the therapeutic window for autophagy activation has not been delineated. Dose-response data addressing whether excessive autophagy increases the risk of autophagic cell death in DCM cardiomyocytes are lacking.

### 5.4 Excessive autophagy activation in genetic DCM

Enomoto et al. (2021) identified a point mutation (Thr320Ala) in the apical domain of the heat shock protein family D member 1 (HSPD1) gene in patients with familial DCM. This residue lies adjacent to the pathogenic Val324Glu mutation in the nbl zebrafish model. Adult nbl zebrafish exhibit a typical DCM phenotype characterized by ventricular wall thinning, chamber dilation, and fibrosis. Their myocardium displays elevated ROS, mitochondrial damage, autophagosome accumulation, and upregulation of autophagy-related genes. HEK293 cells transfected with either HSPD1 or nbl variants exhibit excessive mitophagy activation and exacerbated oxidative stress. These findings implicate ROS-mediated mitophagy hyperactivation driven by HSPD1 apical domain mutations in DCM pathogenesis.

Several caveats warrant consideration. Although this study establishes a pathogenic link connecting the HSPD1 variant to ROS and mitophagy overactivation, whether ROS acts upstream as a cause or downstream as a consequence of enhanced mitophagy remains unresolved. Direct evidence that excessive mitophagy drives cardiomyocyte loss is limited. Moreover, the observations derive from a single family pedigree and a zebrafish model; their generalizability to sporadic DCM awaits further validation.

### 5.5 Impaired autophagic flux in DCM

Pleckstrin homology domain containing family M member 2 (Plekhm2), also termed SifA or kinesin-interacting protein (Skip), governs endosomal trafficking and lysosomal distribution. It participates in endosomal transport through interaction with kinesin-1 and is indispensable for maintaining proper lysosomal positioning within the cooperative regulatory network formed by the ADP ribosylation factor such as GTPase 8 (Arl8) and the biogenesis of lysosome-related organelles complex 1 (BORC) subunits (Jia et al., 2017; Rosa-Ferreira and Munro, 2011). Recessive pathogenic variants in *PLEKHM2* are linked to familial DCM and left ventricular non-compaction (LVNC). Primary fibroblasts from affected individuals exhibit aberrant lysosomal distribution and impaired autophagy (Muhammad et al., 2015). Global *Plekhm2* knockout (PLK2-KO) mice display heightened sensitivity to fasting, with elevated basal AKT phosphorylation and increased LC3-II protein levels. *In vitro*, PLK2-KO cardiac fibroblasts manifest autophagic defects, whereas cardiomyocytes do not, indicating that Plekhm2 loss selectively compromises autophagy in cardiac fibroblasts (Etzion et al., 2024). Furthermore, Plekhm2 deficiency may engage a compensatory mechanism that attenuates susceptibility to angiotensin II (AngII)-induced pathological cardiac hypertrophy in PLK2-KO mice (Etzion et al., 2024).

Bcl-2 associated BAG3 is a critical co-chaperone that governs protein quality control and cellular homeostasis, with particularly prominent roles in the myocardium (Turco, 2023). Pathogenic variants in BAG3 represent a major cause of DCM (Wang et al., 2025). Proteomic, immunoblotting, and myofilament functional analyses were performed on left ventricular tissue from individuals with normal cardiac function, DCM patients lacking the BAG3 (63/380) double variant, and DCM patients harboring this variant. Compared with both normal controls and DCM patients without the variant, carriers of the BAG3 (63/380) double variant exhibit dysregulated autophagy and elevated protein ubiquitination (Martin et al., 2023). Moreover, BAG3 (63/380) variant carriers display distinctive alterations in the expression levels of BAG3-interacting proteins and in their myofilament localization. Notably, the small heat shock protein crystallin alpha B (CRYAB) completely loses its sarcomeric localization in these individuals (Martin et al., 2023).

Heat shock protein 22 (Hsp22), a member of the small heat shock protein family, is predominantly expressed in cardiac and skeletal muscle and exerts essential protective functions against stress-induced myocardial injury (Wu et al., 2020; Depre et al., 2006). Under physiological conditions, Hsp22 knockout in mice leads to progressive cardiac dilation and declining contractile function. Mechanistically, Hsp22 deficiency suppresses BAG3 expression and BAG3-mediated autophagy, resulting in reduced autophagic flux accompanied by disrupted energy homeostasis and oxidative stress (Wu W. et al., 2021).

Heat shock protein family B small member 6 (HSPB6), also known as Hsp20, and its phosphorylation state participate in diverse pathophysiological processes, including myocardial infarction, atherosclerosis, and aortic dissection (Gao et al., 2024; Fan and Kranias, 2011). The human variant *HSPB6* S10F is exclusively detected in patients with DCM. Cardiac expression of this variant in mice markedly suppresses autophagic flux and elevates apoptosis. Mechanistically, HSPB6 tightly interacts with the core autophagy protein Beclin 1 (BECN1), stabilizing its structure and facilitating autophagy initiation. Overexpression of wild-type HSPB6 increases BECN1 abundance and competitively disrupts the BECN1/BCL-2 interaction, thereby further activating autophagy (Liu et al., 2018).

Danon disease is a lethal X-linked disorder caused by pathogenic variants in the lysosomal-associated membrane protein 2 (*LAMP2*) gene. LAMP2 deficiency leads to lysosomal dysfunction and autophagic arrest (Mcnally and Spencer, 2025). Introducing an in-frame deletion of LAMP2 exon 6, which causes human cardiomyopathy, into the mouse *Lamp2* gene (designated L2 [Δ6]) results in a 41-amino acid in-frame deletion. At 40 weeks of age, L2 [Δ6] mice exhibit markedly exacerbated myocardial fibrosis. Immunofluorescence and transmission electron microscopy reveal aberrant lysosomal distribution, massive accumulation of autophagosomes between sarcomeres, and pronounced disruption of cardiomyocyte ultrastructure. Transcriptomic and proteomic analyses demonstrate significant upregulation of genes encoding activators and components linked to autophagy, cardiac hypertrophy, and apoptosis. These findings indicate that impaired autophagy can provoke hypertrophic remodeling and extensive transcriptional reprogramming, which in turn disrupt metabolism, calcium handling, and cell survival (Yadin et al., 2022; Alcalai et al., 2021).

Several mechanistic gaps, however, persist. First, a common downstream pathway uniting these autophagic defects remains elusive. PLEKHM2, BAG3, Hsp22, HSPB6, and LAMP2 operate at distinct steps, including lysosomal positioning, autophagosome formation, autophagic flux, and cargo recognition. Whether these dispersed molecular events converge on shared effectors, such as impaired TFEB nuclear translocation, accumulation of ubiquitinated substrates, or defective mitophagy, to drive the DCM phenotype has not been systematically integrated. Second, the significance of cell type specificity awaits resolution. PLEKHM2 deficiency selectively compromises autophagy in fibroblasts while sparing cardiomyocytes, implying that autophagic defects in genetic DCM may involve non-cardiomyocyte-autonomous effects. How impaired fibroblast autophagy indirectly drives cardiomyocyte dysfunction and DCM progression through paracrine signaling or matrix remodeling requires further exploration. Third, the causal chain linking genetic variants to autophagic phenotypes remains incomplete. Most studies document correlations among mutations, altered autophagic markers, and cardiac dysfunction. Rigorous functional rescue experiments are lacking to define how specific mutations disrupt autophagic flux, whether through reduced induction or impaired degradation, and to quantify the contribution of autophagic defects to cardiomyocyte loss. Fourth, the connection between rare variants and sporadic DCM is tenuous. These findings largely derive from single pedigrees or rare genetic variants; their representativeness for common sporadic DCM and translational value await further assessment.

In summary, advancing the study of autophagic dysfunction in genetic DCM requires a transition from gene–phenotype correlation toward mechanistic network elucidation. Future efforts should prioritize defining the common molecular nodes at which distinct mutations converge to impair autophagy, clarifying the pathological contribution of non-cardiomyocyte autophagy, and pinpointing the precise steps at which autophagic flux is obstructed. Such insights will provide a mechanistic foundation for developing precision interventions that target autophagy in DCM.

### 5.6 Exogenous pharmacological enhancement of autophagy in DCM

The *LMNA* gene encodes A-type lamins. Pathogenic variants in *LMNA* cause a spectrum of disorders collectively termed laminopathies, of which DCM is the most prevalent (Rosario et al., 2023). In the DCM model mice harboring *LMNA* variants, the AKT–mTOR signaling axis is hyperactivated. Treatment with the rapamycin analog temsirolimus delays the progression of cardiac dysfunction in these animals (Choi et al., 2012; Choi and Worman, 2013). Investigation of a DCM patient carrying a heterozygous *LMNA* p. S143P variant revealed markedly increased *LMNA* transcription and mRNA expression in primary fibroblasts. The mutant p. S143P lamin A/C protein substantially suppresses ubiquitin-proteasome system (UPS) activity, leading to nuclear accumulation of K48-linked ubiquitin chains. In response, cells mount a compensatory upregulation of autophagy that selectively degrades K48-ubiquitinated lamin A/C to preserve proteostasis (West et al., 2022). Administration of 4-phenylbutyrate (4-PBA) to patient cells restores both UPS activity and autophagic function, thereby efficiently promoting the clearance of aberrant proteins (West et al., 2022).

DOX blocks autophagic flux in cardiomyocytes by inhibiting lysosomal acidification and function. The resultant accumulation of autolysosomes may elevate ROS production and exacerbate myocardial injury (Li et al., 2016). Transcription factor EB (TFEB) serves as a master regulator of lysosomal gene transcription and function. Atorvastatin (ATO) increases TFEB protein levels and elevates the ratio of lysosomal-associated membrane protein 2 (LAMP2) to microtubule associated protein 1 light chain three beta (LC3B) in the myocardium of DOX-induced DCM mice, suggesting that ATO enhances lysosomal function and autophagic activity through TFEB upregulation (Jiang et al., 2022).

Although these observations underscore the therapeutic potential of autophagy modulation, critical gaps persist. 4-PBA concurrently affects both the UPS and autophagy; the relative contribution of each pathway to its cardioprotective effects has not been dissected through single-pathway intervention experiments. Whether TFEB upregulation results from direct ATO action or off-target effects independent of its lipid-lowering properties also remains undefined. Both agents have been validated in single model systems, and their efficacy in other DCM subtypes and in human disease awaits further evaluation.

### 5.7 Biphasic nature of autophagy activation in DCM

Autophagy is activated during DCM progression. Although moderate additional stimulation is considered beneficial, autophagy is a tightly regulated process whose excessive activation can exacerbate disease pathology (Enomoto et al., 2021). Xie et al. (2013) reported that rapamycin activates autophagy in a rat DCM model induced by cardiac myosin immunization. Autophagic vacuole formation was observed, accompanied by reduced phosphorylation of p70S6 kinase (p70S6K) and eukaryotic translation initiation factor 4E binding protein 1 (4E-BP1), and an increased LC3-II/LC3-I ratio. Low-dose (1 mg/kg/day) and medium-dose (2 mg/kg/day) rapamycin improved left ventricular ejection fraction (LVEF) in this model. High-dose treatment (4 mg/kg/day), however, increased mortality (Xie et al., 2013). The narrow therapeutic window underscores the considerable challenge of implementing autophagy-activation strategies in the clinical management of DCM patients. Table 6 summarizes the intervention targets, experimental models, and evidence types of ferroptosis.

**FIGURE 5:**
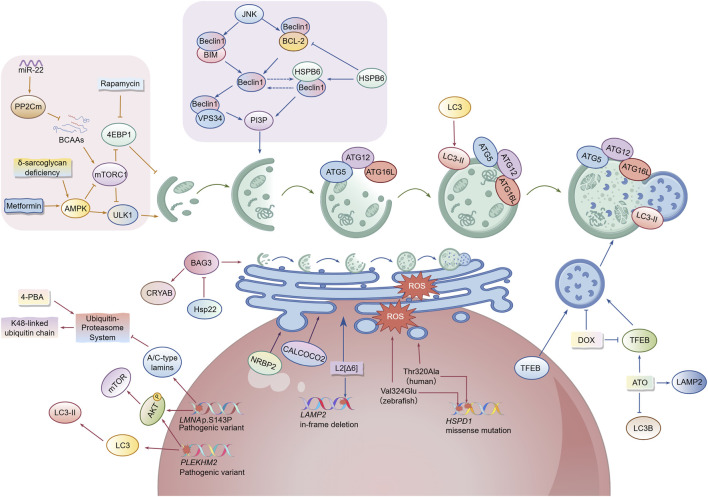
Signaling of autophagy in dilated cardiomyopathy. (1) Initiation and vesicle nucleation. AMPK activates and inhibits mTORC1, relieving mTORC1-mediated suppression of the ULK1 complex to initiate autophagy. Rapamycin inhibits the mTOR–4E-BP1 pathway, thereby activating autophagy. miR-22 upregulates PP2Cm, which promotes BCAA catabolism, attenuates mTOR activation, and enhances autophagy. δ-Sarcoglycan deficiency elevates AMPK expression, suppresses mTOR, and concurrently augments autophagy. Metformin activates AMPK/mTOR signaling and increases autophagic flux. (2) Vesicle elongation. Activated JNK phosphorylates BCL-2 and BIM, disrupting Beclin1/BCL-2 and Beclin1/BIM complexes to liberate Beclin1. Free Beclin1 activates and associates with VPS34 to form a complex that generates PI3P, driving phagophore elongation. HSPB6 upregulates BECN1 expression and competitively disrupts the BECN1–BCL-2 interaction, further enhancing autophagy. (3) Autophagosome formation. ATG5, ATG12, and ATG16L1 act in concert to process and lipidate LC3-I into its membrane-bound LC3-II form, which anchors to the autophagosomal membrane and completes autophagosome formation. (4) Autolysosome formation. TFEB enhances lysosomal function. ATO upregulates TFEB protein levels and increases the LAMP2 to LC3B ratio, thereby promoting autophagic clearance. (5) Autophagic dysregulation caused by genetic variants. The human HSPD1 apical domain variant Thr320Ala and the corresponding zebrafish Val324Glu variant drive excessive mitophagy through ROS. PLEKHM2 loss-of-function variants elevate AKT phosphorylation and LC3-II levels, impairing autophagy in primary fibroblasts. In-frame deletion of LAMP2 exon 6 (L2 [Δ6]) results in autophagic hyperactivation. LMNA pathogenic variants hyperactivate AKT–mTOR signaling and profoundly suppress UPS activity, causing nuclear accumulation of K48-linked ubiquitin chains. 4-PBA treatment restores both UPS activity and autophagic function. (6) Additional regulatory modules. Hsp22 promotes BAG3 expression to maintain autophagic homeostasis; BAG3, in turn, sustains CRYAB levels. Altered expression of the autophagy-related genes NRBP2 and CALCOCO2 correlates with autophagic activity in cardiomyocytes.

Autophagy is broadly engaged in DCM, yet its functional outcome is highly context dependent. Moderate, flux-competent autophagy clears toxic proteins and damaged organelles, exerting cardioprotective effects that correlate with a favorable prognosis. Conversely, autophagic dysregulation precipitated by specific genetic variants or pathological insults, whether through hyperactivation or impaired flux, drives cardiomyocyte death and disease progression. Extensive crosstalk exists between autophagy and other PCD modalities, including apoptosis and necroptosis. Impaired autophagic flux can provoke accumulation of misfolded proteins and endoplasmic reticulum stress, thereby triggering apoptosis, whereas excessive autophagy can directly induce cell death and coexist with oncosis, generating a complex “death network.” Therapeutically, precise restoration of autophagic homeostasis, rather than wholesale activation or inhibition, is essential. Targeting upstream regulatory nodes such as mTOR, AMPK, or TFEB, or restoring lysosomal function, represents a potential strategy. The dose-dependent effects of rapamycin underscore the narrow therapeutic window for autophagy modulation. Table 5 summarizes the intervention targets and experimental models of autophagy. Figure 5 depicts autophagy signaling in dilated cardiomyopathy.

**TABLE 5:** Autophagy intervention targets and experimental models.

Autophagy intervention target	Therapeutic strategy or drug	Brief description of core mechanism	Experimental model
mTOR (Jin et al., 2020)	Rapamycin (low to medium dose)	Inhibits the mTOR–4EBP1 pathway, moderately activates autophagy, and improves cardiac function	BALB/c mice with EAM-DCM induced by porcine cardiac myosin immunization
mTOR (Choi et al., 2012; Choi and Worman, 2013)	Temsirolimus (rapamycin analog)	Suppresses hyperactivated AKT–mTOR signaling and delays cardiac functional decline in LMNA-related DCM	LMNA variant transgenic mouse model
AMPK/mTOR (Kanamori et al., 2019)	Metformin	Activates AMPK, promotes autophagic flux, and improves cardiac function in δ-sarcoglycan-deficient DCM mice	δ-sarcoglycan-deficient DCM mice (32 weeks old)
miR-22 (Caragnano et al., 2019)	Downregulation of miR-22	Restores PP2Cm expression, promotes BCAA catabolism, and ameliorates mTOR-mediated autophagic inhibition and TFEB nuclear localization	Myocardial tissue from 62 DCM heart transplant recipients plus primary cells (miR-22 downregulation reverses molecular defects)
TFEB (Jiang et al., 2022)	Atorvastatin	Upregulates TFEB expression, enhances lysosomal function and autophagic activity, and ameliorates DOX-induced autophagic flux blockade	DOX-induced cardiomyopathy mouse model
UPS/Autophagy (West et al., 2022)	4-PBA	Restores ubiquitin-proteasome activity and autophagic function, promoting clearance of aberrant proteins linked to LMNA variants	Primary fibroblasts from a DCM patient harboring a heterozygous LMNA p.S143P variant
HSPB6 (Liu et al., 2018)	Overexpression of wild-type HSPB6	Stabilizes BECN1, competitively disrupts the BECN1–BCL-2 interaction, and activates autophagic flux	Mouse hearts overexpressing wild-type *HSPB6* or the *HSPB6* S10F variant (identified in DCM patients)

## 6 Ferroptosis in DCM

Iron is an essential trace element for nearly all living organisms. Its facile electron exchange renders free iron highly reactive and potentially toxic (Ganz, 2013). Iron participates in fundamental biological processes, including oxygen transport, energy metabolism, and DNA synthesis (Zeidan et al., 2021). Maintenance of iron homeostasis is therefore critical for normal cellular function (Sangkhae et al., 2023; Long et al., 2020; Mu et al., 2021). Ferroptosis is a form of programmed cell death driven by iron-dependent lipid peroxidation, ultimately resulting from failure of cellular antioxidant systems, particularly the glutathione peroxidase 4 (GPX4) pathway. Although formally named in 2012, ferroptosis represents an evolutionarily ancient and widespread cell death modality (Dixon et al., 2012).

### 6.1 Prominent ferroptosis in DCM

The role of cardiomyocyte ferroptosis in cardiovascular disease has garnered increasing attention. Evidence implicates ferroptosis in diverse cardiac pathologies, including cardiomyopathy, myocardial ischemia, and heart failure (Wu X. et al., 2021; Sheng et al., 2023; Wu et al., 2024). In myocardial samples from patients with DCM, substantial cardiomyocyte loss and lipid peroxidation are also evident (Nakamura et al., 2002; She et al., 2025).

### 6.2 Molecular mechanisms of ferroptosis in DCM

In cardiomyocytes, the canonical ferroptosis regulatory mechanism centers on the system Xc^−^ cystine/glutamate antiporter, which imports cystine. Cystine is subsequently reduced to cysteine for the synthesis of glutathione (GSH) (Dixon and Olzmann, 2024). GSH functions as a potent reductant and serves as an essential cofactor for GPX4. GPX4 reduces phospholipid hydroperoxides (PLOOHs) generated within cells to their corresponding alcohols (PLOHs). Regeneration of oxidized glutathione (GSSG) is catalyzed by glutathione reductase (GSR) in a process dependent on NADPH/H^+^ (Jiang et al., 2021). GPX4 is highly expressed in the heart (Knopp et al., 1999). Cardiomyocyte-specific deletion of *GPX4* promotes iron overload and ferroptosis (Duan et al., 2026), directly leading to DCM-like pathological remodeling (Guo X. et al., 2025). Conversely, cardiomyocyte-derived GPX4 attenuates myocardial injury and mitochondrial dysfunction, thereby alleviating long-term ventricular remodeling (Zhong et al., 2026). Ferroptosis suppressor protein 1 (FSP1) is another critical ferroptosis regulator that operates independently of the GPX4 pathway. FSP1 reduces ubiquinone to ubiquinol, directly scavenging free radicals to terminate lipid peroxidation chain reactions. It also reduces α-tocopherol radicals back to α-tocopherol, restoring the activity of this most effective chain-breaking antioxidant within lipid membranes (Dixon and Olzmann, 2024). Additional signaling pathways, including AMPK and nuclear factor erythroid 2-related factor 2 (Nrf2), also participate in regulating cellular ferroptosis. AMPK activation limits ferroptosis in stressed cardiomyocytes by modulating lipid metabolism, whereas Nrf2 governs the expression of antioxidant enzymes that protect against ferroptotic injury. When iron or energy metabolism becomes dysregulated in cardiomyocytes, excessive iron or ROS accumulation triggers lipid peroxidation, culminating in ferroptotic cell death (Jiang et al., 2021; Li et al., 2020).

#### 6.2.1 Iron dysregulation and iron overload

Iron homeostasis constitutes a fundamental physiological requirement for maintaining the structural and functional integrity of cardiomyocytes, governed by the dynamic balance among iron uptake, storage, efflux, and utilization (Lakhal-Littleton, 2019). Disruption of iron metabolism and consequent iron overload precipitate oxidative stress and mitochondrial damage, thereby driving ferroptosis (Mancardi et al., 2021; Mousavi-Aghdas et al., 2024). Under pathological conditions, inflammation-driven dysregulation of the hepcidin–ferroportin axis promotes aberrant iron accumulation within cardiomyocytes (Awashra et al., 2026). Cardiomyocyte-specific GPX4 deletion directly triggers iron overload through the BTB domain and CNC homolog 1 (Bach1)/heme oxygenase 1 (HO-1) signaling axis, thereby promoting cardiac ferroptosis and eliciting a DCM phenotype (Guo X. et al., 2025). Excess ferrous iron (Fe^2+^) enters cardiomyocytes predominantly *via* L-type calcium channels (LTCCs). This free iron fuels ferroptosis through the Fenton reaction, which generates highly reactive hydroxyl radicals (·OH) that attack polyunsaturated fatty acids (PUFAs) within cellular membranes, initiating a self-amplifying chain reaction of lipid peroxidation (Murphy and Oudit, 2010). The heart is exquisitely vulnerable to this process owing to its high oxygen consumption, abundant mitochondria, and enrichment in PUFA-containing phospholipids (Schwarz et al., 2014; Cho et al., 2025). Clinically, primary hemochromatosis, primarily associated with HFE gene variants, and secondary iron overload resulting from repeated blood transfusions both represent significant triggers of DCM. Affected patients frequently exhibit myocardial fibrosis and impaired systolic function. With respect to diagnosis and management, cardiac magnetic resonance imaging (MRI) serves as a sensitive noninvasive modality for evaluating myocardial iron deposition. Early phlebotomy and iron chelation therapy can reverse myocardial injury and delay DCM progression (Murphy and Oudit, 2010).

Solute carrier family 40 member 1 (SLC40A1), the sole known mammalian iron efflux protein, is a transmembrane transporter responsible for exporting ferrous iron (Fe^2+^) from the intracellular compartment to the extracellular milieu (Debbiche et al., 2024). Its expression is tightly governed by the liver-derived hormone hepcidin (Uguen et al., 2023). Elevated systemic iron levels promote hepcidin binding to SLC40A1, triggering its internalization and lysosomal degradation, thereby restricting iron export and preserving whole-body iron homeostasis (Nemeth et al., 2004; Link et al., 2021). Within the heart, SLC40A1 is expressed in cardiomyocytes, where it plays a pivotal role in cellular iron release and is essential for maintaining normal cardiac function (Lakhal-Littleton et al., 2015).

Aberrant SLC40A1 expression disrupts intracellular iron homeostasis and contributes to cardiac pathology through bidirectional mechanisms. Both gain-of-function (or upregulation) and loss-of-function (or downregulation) states mediate distinct pathological processes (Uguen et al., 2024; Zhang Y. et al., 2024). Genetic evidence substantiates the clinical relevance of SLC40A1 in cardiomyopathy. Gain-of-function variants in SLC40A1 cause hemochromatosis type 4, also termed ferroportin disease, a condition characterized by systemic iron overload that can progress to DCM (Li Y. et al., 2024; Pietrangelo, 2025). These genetic findings underscore the importance of SLC40A1-mediated iron export in preserving cardiac iron homeostasis.

In the context of ferroptosis, SLC40A1 has been identified as a critical regulatory node (Zhang Y. et al., 2024). Mechanistic studies reveal that SLC40A1 expression is controlled by the AKT/NRF2 signaling axis, and its downregulation promotes intracellular iron accumulation and ferroptosis induction (Zheng et al., 2025). Epigenetic regulation of SLC40A1 by DNA methyltransferase 3B (DNMT3B) also modulates ferroptosis susceptibility (Chen et al., 2024). It must be acknowledged, however, that direct evidence linking SLC40A1 dysregulation to ferroptosis specifically in DCM remains limited. Current evidence derives largely from ischemic heart disease models (Feng et al., 2024), cancer studies (Mansuer et al., 2024), or systemic iron overload disorders (Li Y. et al., 2024). Future investigations should directly examine the role of SLC40A1 in DCM-associated ferroptosis and explore its potential as a therapeutic target.

#### 6.2.2 Ferritinophagy- and mTOR-mediated ferroptosis–autophagy crosstalk

Ferritinophagy is a selective autophagic process mediated by nuclear receptor coactivator 4 (NCOA4). NCOA4 recognizes and binds ferritin, the principal intracellular iron storage protein complex, targeting it to autophagosomes for subsequent lysosomal degradation and release of free iron (Mancias et al., 2014). This process expands the intracellular labile iron pool (LIP). Excess free iron subsequently drives the Fenton reaction, generating hydroxyl radicals that initiate lipid peroxidation chain reactions and ultimately promote ferroptosis (Liu M. Z. et al., 2022). NCOA4 expression and activity are tightly regulated by intracellular iron levels (Kuno and Iwai, 2023), oxidative stress (Liang et al., 2025), and multiple signaling pathways, including mTOR (Wu et al., 2025), positioning ferritinophagy as a critical nexus connecting iron metabolism to ferroptosis.

Accumulating evidence implicates ferritinophagy as a key mechanism driving cardiomyocyte ferroptosis across diverse cardiac pathologies. In a pressure overload-induced heart failure mouse model, ferritinophagy activation promotes cardiomyocyte ferroptosis, whereas NCOA4 knockdown attenuates iron accumulation and lipid peroxidation (Ito et al., 2021). In diabetic cardiomyopathy, downregulation of the prorenin receptor alleviates cardiac pathology by suppressing NCOA4-mediated ferritinophagy (Zhang X. et al., 2024), underscoring the translational potential of targeting this axis. Furthermore, in myocardial ischemia-reperfusion injury, sentrin-specific protease 2 (SENP2)-mediated deSUMOylation of NCOA4 inhibits ferritinophagy-dependent ferroptosis, thereby facilitating functional recovery (Xue et al., 2025). Although these findings derive from models of heart failure, diabetic cardiomyopathy, and ischemia-reperfusion injury, the NCOA4-dependent ferritinophagy mechanism exhibits cross-disease conservation, providing a compelling rationale for exploring this pathway in DCM. Direct evidence for ferritinophagy in DCM, however, remains sparse and awaits further validation.

The mechanistic target of rapamycin, particularly mTOR complex 1 (mTORC1), serves as a master regulator of autophagy (Valvezan and Manning, 2019) and has emerged as a critical signaling node integrating iron metabolism with ferroptosis (Jee et al., 2025). mTORC1 activity is tightly governed by nutrient availability and cellular stress. Under basal conditions, mTORC1 inhibition induces autophagy, including ferritinophagy, which liberates free iron, triggers lipid peroxidation, and thereby promotes ferroptosis (Jee et al., 2025; Koshizuka et al., 2025). Conversely, sustained mTORC1 hyperactivation is equally detrimental: it downregulates ferritin expression, expands the labile iron pool, and exacerbates ferroptosis (Jee et al., 2025). This establishes a bidirectional positive feedback loop between mTORC1 signaling and ferroptotic death.

In cardiac pathology, mTOR is frequently hyperactivated. Under these circumstances, moderate mTOR inhibition corrects this excessive activity and restores autophagic flux to physiological levels, thereby alleviating myocardial injury (Duan et al., 2024). This scenario does not contradict the observation that mTOR inhibition under basal conditions promotes ferroptosis; the former aims to reestablish homeostasis, whereas the latter disrupts it. Notably, cardiomyocyte-specific deletion of mTOR in adult mice precipitates lethal DCM characterized by apoptosis, aberrant autophagy, and disrupted mitochondrial architecture, directly demonstrating the essential requirement for intact mTOR signaling for preserving normal cardiac structure and function. Based on these lines of evidence, mTOR can be viewed as a key upstream regulator linking cardiomyocyte autophagy and ferroptosis. The net effect is highly context-dependent, governed by the underlying pathological state and the degree of mTOR modulation.

Collectively, NCOA4-mediated ferritinophagy and its upstream mTOR signaling axis constitute a core network regulating cardiomyocyte ferroptosis. Although direct evidence currently derives primarily from models of heart failure, diabetic cardiomyopathy, and ischemia–reperfusion injury, these findings imply potential relevance of this mechanism in DCM. Future studies should directly validate the role of the ferritinophagy–mTOR axis in DCM models and explore its clinical translational value as a therapeutic target.

### 6.3 Epigenetic regulation of ferroptosis in DCM

#### 6.3.1 RNA and DNA methylation regulating ferroptosis-related genes

RNA methylation, particularly N6-methyladenosine (m^6^A) modification, represents the most prevalent reversible epitranscriptomic mark on eukaryotic mRNA. By governing RNA stability, splicing, and translational efficiency, it participates in the pathophysiology of diverse cardiovascular disorders (Liu et al., 2026), and its role in DCM has attracted increasing attention. m^6^A modification modulates expression of the ferroptosis-associated gene solute carrier family 7 member 11 (SLC7A11) through enzymes including methyltransferase like 3 (METTL3) and METTL14, thereby balancing cardiomyocyte death and survival pathways while improving mitochondrial function and attenuating oxidative stress to indirectly suppress ferroptosis (Zhang et al., 2025). Consequently, small-molecule inhibitors targeting m^6^A regulatory enzymes, together with gene-editing approaches, may represent promising avenues for personalized DCM therapy.

In contrast, direct evidence for DNA methylation in DCM remains sparse, although mechanistic insights from other disease models offer a valuable framework for future investigation. The GPX4 promoter is enriched in CpG islands and is highly susceptible to DNA methylation (Meng et al., 2024). Whether DNA methylation participates in cardiomyocyte ferroptosis in DCM by modulating GPX4 or SLC40A1 expression currently lacks direct evidence and awaits rigorous investigation.

#### 6.3.2 Role of histone deacetylases in DCM-associated ferroptosis and apoptosis

Histone deacetylases are a core class of epigenetic enzymes that remove acetyl groups from lysine residues on histones and non-histone proteins, thereby modulating chromatin architecture and gene transcription (Yang and Seto, 2008). Mammalian HDACs comprise 18 isoforms grouped into four classes: class I (HDAC1, HDAC2, HDAC3, and HDAC8), class II (IIa: HDAC4, HDAC5, HDAC7, and HDAC9; IIb: HDAC6 and HDAC10), class III sirtuins, and class IV (HDAC11) (Haberland et al., 2009).

HDACs play pivotal roles in cardiomyocyte survival and death in DCM through the regulation of apoptosis-related gene expression. Dual deletion of HDAC1 and HDAC2 in mice precipitates a DCM phenotype characterized by arrhythmias, neonatal lethality, and upregulation of skeletal muscle-specific contractile proteins and calcium channel-related genes, underscoring the essential requirement for class I HDACs in preserving normal cardiac structure and function (Montgomery et al., 2007). In DCM animal models, HDAC6 expression is markedly elevated. HDAC6 knockdown attenuates DCM-induced ventricular remodeling and cardiac injury through a dual mechanism: it suppresses NLRP3 inflammasome activation to reduce cardiomyocyte pyroptosis while simultaneously promoting autophagy to reduce cardiomyocyte apoptosis (Pang et al., 2023). In *BAG3*-knockout DCM mice, the HDAC6-specific inhibitor TYA-018 alleviates sarcomeric injury, improves mitochondrial function, decreases cardiomyocyte apoptosis, elevates left ventricular ejection fraction, and reduces left ventricular internal diastolic diameter, further supporting HDAC6 as a therapeutic target in DCM (Yang et al., 2022). Additionally, HDAC5 has been identified as a critical epigenetic regulator in *TTN*tv-associated DCM; HDAC5 knockdown reverses *TTN* deficiency-induced cardiac dysfunction and profibrotic gene expression (Hasan et al., 2025).

The therapeutic potential of HDAC inhibitors (HDACi) in DCM has been preliminarily validated. Class I and class II HDAC inhibitors improve acetylation status and energetic state in human myocardial mesenchymal stem cells (hmMSCs) derived from DCM patients, activate cardiac transcription factors (NK2 homeobox 5, NKX2-5; homeodomain-only protein, HOPX; GATA binding protein 4, GATA4; and myocyte enhancer factor 2C, Mef2C), and upregulate myocardial structural proteins (cardiac troponin T and α-cardiac actin), suggesting that HDACi may serve as novel therapeutic modulators for DCM (Mikšiūnas et al., 2024). The class IIa HDAC inhibitor TMP-195 reverses *TTN* deficiency-induced gene expression dysregulation, restores cardiac functional gene expression, and suppresses profibrotic gene signatures (Hasan et al., 2025), highlighting the considerable promise of HDAC inhibitors in precision therapy for DCM. Future investigations should further delineate the isoform-specific contributions of distinct HDACs to ferroptosis and apoptosis in DCM and explore the clinical translational prospects of isoform-selective HDAC inhibitors in DCM management.

#### 6.3.3 Regulation of ferroptosis by non-coding RNAs in DCM cardiomyocytes

Diverse non-coding RNAs, including miRNAs, lncRNAs, and circRNAs, participate in cardiomyocyte ferroptosis through post-transcriptional regulation and hold promise as both diagnostic biomarkers and therapeutic targets (Liu Y. et al., 2023). The ceRNA hypothesis provides a unifying framework for integrating the regulatory interplay among these RNA species and has been widely adopted in DCM research.

At the miRNA level, analyses of DCM myocardial microarray datasets from the GEO have constructed transcription factor–mRNA–miRNA regulatory networks and identified ferroptosis-associated miRNA modules (Wang Z et al., 2022), miRNAs also contribute to apoptosis, autophagy, and mitochondrial dysfunction in DCM cardiomyocytes (Alonso-Villa et al., 2022). At the lncRNA level, ceRNA networks built from miRNA microarray data of DCM heart samples have identified multiple functional lncRNAs targeting miR-144/451 and miR-21 (Tao et al., 2019). Mechanistic studies further demonstrate that lncRNA zinc finger antisense 1 (ZFAS1) acts as a ceRNA to sponge miR-150-5p, thereby downregulating cyclin D2 (CCND2) and promoting cardiomyocyte ferroptosis and disease progression (Ni et al., 2021). At the circRNA level, circPIK3C2A enhances transferrin receptor (TFRC) expression by sponging miR-31-5p, forming a circPIK3C2A/miR-31-5p/TFRC axis that drives iron overload-mediated ferroptosis (Miao et al., 2020). Given the frequent occurrence of myocardial iron dysregulation in DCM, this axis carries potential pathological significance. High-throughput sequencing of plasma exosomes from DCM patients with chronic heart failure has also identified 49 differentially expressed circRNAs implicated in histone acetyltransferase activity and vesicular trafficking (Xu et al., 2024). Collectively, non-coding RNAs regulate cardiomyocyte ferroptosis in DCM through multilayered ceRNA networks. Their differential expression profiles offer candidate molecular signatures for noninvasive diagnosis and prognostic assessment.

### 6.4 Interplay between immune heterogeneity and ferroptosis in DCM

Studies in lung adenocarcinoma and osteosarcoma have established a close association between immune heterogeneity and ferroptosis (Zhang et al., 2021; Lei et al., 2021). DCM encompasses multiple subtypes with substantial inter-subtype heterogeneity (Verdonschot et al., 2021). From an immune heterogeneity perspective, Lu et al. (2023) demonstrated that ferroptosis modulates the immune microenvironment in patients with DCM. The ferroptosis-related gene OTU deubiquitinase 1 (OTUD1) contributes to immune heterogeneity through the regulation of B cells and dendritic cells.

### 6.5 Ferroptosis triggered by inherited metabolic defects in DCM

In primary carnitine deficiency-associated DCM, pathogenic variants in the solute carrier family 22 member 5 (SLC22A5) gene cause functional deficiency of the organic cation transporter novel type 2 (OCTN2), impairing carnitine uptake. This precipitates lipid accumulation, mitochondrial structural abnormalities, and metabolic remodeling in cardiomyocytes, ultimately triggering iron-dependent and lipid-dependent ferroptosis (Loos et al., 2023).

She et al. (2025) investigated a DCM mouse model with cardiomyocyte-specific overexpression of mammalian STE20-like kinase 1 (Mst1). Transcriptomic profiling, mitochondrial Fe^2+^ quantification, lipid peroxidation assays, and mitochondrial morphological analysis confirmed prominent ferroptosis in these cardiomyocytes. Mst1 overexpression directly activates the Hippo pathway, preventing the downstream effector Yes-associated protein (YAP) from engaging its canonical transcriptional partner, TEA domain transcription factor (TEAD). Instead, YAP forms an inhibitory complex with Yin Yang 1 (YY1) that targets the NFS1 gene promoter and suppresses its transcription. NFS1 is the rate-limiting enzyme for iron–sulfur cluster (ISC) biosynthesis. NFS1 downregulation impairs ISC production, triggering an “iron starvation response” characterized by iron regulatory protein 1 (IRP1) nuclear translocation, upregulation of the iron uptake gene transferrin receptor (TFRC), and downregulation of the iron storage gene ferritin heavy chain 1 (Fth1). This cascade results in massive mitochondrial Fe^2+^ accumulation. Excess mitochondrial Fe^2+^ induces a burst of ROS and glutathione depletion, initiating lipid peroxidation, culminating in cardiomyocyte ferroptosis, and driving DCM progression. Administration of the ferroptosis inhibitor ferrostatin-1 (Fer-1) or restoration of NFS1 expression via adeno-associated virus serotype 9 (AAV9) reverses iron overload and lipid peroxidation and improves cardiac function.

### 6.6 Ferroptosis in drug-induced DCM and therapeutic interventions

Ferroptosis constitutes a key pathological mechanism in DOX-induced DCM. DOX disrupts cardiomyocyte redox homeostasis, perturbs both cytosolic and mitochondrial iron metabolism, and damages mitochondrial structure and function, directly triggering cardiomyocyte ferroptosis (Tadokoro et al., 2020; Shao et al., 2025). This process is accompanied by TGF-β1-mediated myocardial fibrosis and macrophage-driven inflammation, establishing a synergistic “ferroptosis–inflammation–fibrosis” injury network that culminates in left ventricular dilation and progressive cardiac functional decline (Haesen et al., 2024). The vitamin B6 derivative pyridoxamine (PM) exerts cardioprotective effects through coordinated multi-pathway actions centered on precise modulation of ferroptotic signaling. At the genetic and functional levels, PM reverses DOX-induced redox imbalance, restores cellular and mitochondrial iron handling, and alleviates mitochondrial structural damage, thereby suppressing ferroptosis initiation at its source. Additionally, PM inhibits TGF-β1-associated fibrotic remodeling and macrophage-mediated inflammatory responses, effectively disrupting the feed-forward “ferroptosis-inflammation” loop (Haesen et al., 2024).

Moreover, DOX treatment induces degradation of the Parkinson disease-associated deglycase (Park7) in a p53-dependent manner. Under physiological conditions, Park7 associates with iron-sulfur clusters and functions as an upstream regulator of iron homeostasis. Park7 degradation impairs iron–sulfur cluster-mediated aconitase activity and disrupts expression of iron homeostasis-related proteins, leading to dysregulation of both cytosolic and mitochondrial iron balance. Importantly, DOX-induced iron dyshomeostasis differs mechanistically from secondary iron overload triggered by ferric ammonium citrate (FAC), indicating a distinct regulatory mechanism. Park7 antagonizes iron overload by modulating transcription of the iron regulatory protein family while simultaneously blocking mitochondrial iron uptake. Cardiomyocyte-specific p53 knockout or Park7 overexpression restores iron-sulfur cluster activity and iron homeostasis, thereby suppressing ferroptosis (Pan et al., 2023). Collectively, the p53-Park7 signaling axis represents a potential therapeutic target in DOX-induced cardiomyopathy.

### 6.7 Distinct ferroptotic signatures across cardiomyopathy subtypes

Bioinformatic analyses reveal subtype-specific ferroptosis-related genes in hypertrophic cardiomyopathy (HCM), including activating transcription factor 3 (ATF3), lysophosphatidylcholine acyltransferase 3 (LPCAT3), and solute carrier family 1 member 5 (SLC1A5). In contrast, STAT3 emerges as a central ferroptosis regulator in DCM, indicating mechanistic divergence between these conditions. Three shared core genes, periostin (POSTN), insulin-like growth factor binding protein 5 (IGFBP5), and fibromodulin (FMOD), may indirectly influence ferroptosis-associated metabolism through the growth hormone pathway, offering potential targets for common therapeutic intervention (Wang Z. et al., 2022).

Ferroptosis is robustly activated across diverse DCM models, including genetic- and drug-induced forms. Its core mechanism involves the collapse of the system Xc^−^–GPX4 antioxidant axis and dysregulation of the FSP1-CoQ10 shunt, culminating in lethal lipid peroxidation. Key molecular crosstalk events underscore that ferroptosis does not operate in isolation. It can be triggered by upstream signals, such as the Hippo pathway impairing iron metabolism through NFS1 suppression, and by epigenetic modifications such as m^6^A. Simultaneously, its products, including lipid peroxides, and its consequences, such as the release of cellular contents, potently drive inflammation and fibrosis, establishing a vicious “ferroptosis–inflammation–fibrosis” cycle. This cycle is further shaped by immune microenvironment heterogeneity, exemplified by OTUD1-mediated regulation. These interactions reveal a coordinated mechanism linking cardiomyocyte loss with adverse tissue remodeling in DCM. Therapeutically, targeting core ferroptotic nodes by restoring GPX4 activity, activating FSP1, or employing iron chelators and antioxidants such as pyridoxamine offers pleiotropic potential to concurrently mitigate cell death, inflammation, and fibrosis.

### 6.8 Therapeutic potential of iron chelators in DCM

Iron chelators, which directly modulate iron homeostasis, have demonstrated potential clinical value. Currently approved iron chelators include deferoxamine, deferiprone, and deferasirox (Fernandes, 2012). Deferoxamine is administered intravenously or subcutaneously and requires prolonged continuous infusion owing to poor intestinal absorption, which limits long-term adherence (Salimi et al., 2019). Deferiprone exhibits high oral bioavailability and a small molecular size, enabling effective penetration of the cardiomyocyte membrane for direct intracellular iron clearance (Glickstein et al., 2006). Deferasirox, a once-daily oral iron chelator, has established efficacy in maintaining negative iron balance and reducing hepatic iron concentration (Chaudhary and Pullarkat, 2013).

In iron overload cardiomyopathy, the efficacy of iron chelators in reversing cardiac functional decline is well established (Pennell et al., 2006; Cooray et al., 2018). A randomized controlled trial enrolling 61 patients with β-thalassemia major demonstrated that deferiprone monotherapy significantly outperformed deferoxamine in improving right ventricular ejection fraction (RVEF) and right ventricular end-systolic volume (RVESV) (Smith et al., 2011). Deferiprone doses exceeding 80 mg/kg/day rapidly clear myocardial iron, reverse iron overload-associated cardiomyopathy, and preserve normal iron reserves (Kolnagou et al., 2008). Small-scale iron chelation trials in heart failure patients confirm that both deferoxamine monotherapy and combined deferoxamine-based therapy effectively improve left ventricular ejection fraction (LVEF) and myocardial T2 values (Porter et al., 2013). Combined therapeutic strategies are gaining attention for severe cases. Deferiprone and deferoxamine exhibit synergistic or additive effects on cardiac iron clearance (Tanner et al., 2007), a phenomenon potentially attributable to the “shuttling” of iron from deferiprone to deferoxamine (Link et al., 2001). In iron-overloaded rats, deferiprone combined with the antioxidant N-acetylcysteine demonstrates superior cardioprotection compared with monotherapy, reflected by reduced cardiac iron deposition and improved mitochondrial function (Wongjaikam et al., 2016).

Direct application of iron chelators in DCM faces considerable challenges. Current evidence derives primarily from systemic iron overload disorders such as β-thalassemia and transfusion-related hemosiderosis. Myocardial iron burden in DCM exhibits marked heterogeneity, and not all patients display significant cardiac iron deposition. Patient selection, therefore, requires cardiac magnetic resonance T2 imaging to identify suitable candidates. Moreover, iron chelators carry potential toxicities: deferoxamine can cause renal injury, deferiprone may provoke agranulocytosis, and deferasirox is also associated with renal impairment. The risk of adverse effects warrants particular vigilance when these agents are used in the absence of overt iron overload (Botzenhardt et al., 2017).

**FIGURE 6:**
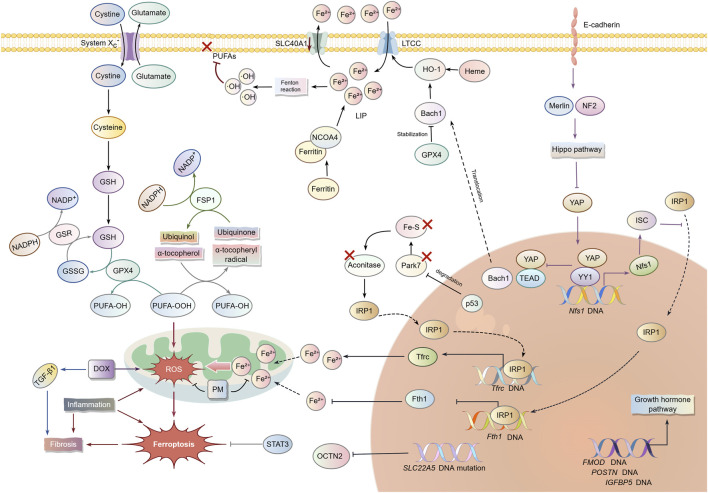
Signaling of ferroptosis in dilated cardiomyopathy. Core ferroptosis pathway. The system Xc^−^ antiporter imports cystine and exports glutamate. Intracellular cystine is reduced to cysteine for GSH synthesis. GSH activates GPX4, which reduces toxic PLOOHs to benign PLOHs, thereby suppressing lipid peroxidation. Oxidized glutathione (GSSG) is regenerated to GSH by GSR using electrons from NADPH, completing the cycle. FSP1 reduces ubiquinone to ubiquinol, which terminates lipid peroxidation chain reactions. FSP1 also reduces α-tocopherol radicals, restoring the antioxidant activity of α-tocopherol. Regulatory network of ferroptosis in DCM. (1) Mst1/Hippo–YAP/YY1 axis. Hippo pathway kinase Mst1 inhibits its downstream effector YAP. Inactivated YAP instead forms a complex with transcription factor YY1 that binds and represses the NFS1 promoter. NFS1 is the rate-limiting enzyme for mitochondrial ISC synthesis. Its suppression triggers an iron starvation response: IRP1 translocates to the nucleus, upregulating the iron uptake gene TFRC and downregulating the iron storage gene Fth1, ultimately causing mitochondrial Fe^2+^ overload. Excess iron drives the Fenton reaction, generating ROS, triggering lipid peroxidation and GSH depletion, and driving ferroptosis. (2) Inherited metabolic defects. SLC22A5 pathogenic variants cause loss of carnitine transporter OCTN2 function, disrupting cardiomyocyte lipid metabolism and directly triggering ferroptosis. (3) Drug induction and intervention. DOX disrupts iron homeostasis and mitochondrial function, acting as a key ferroptosis trigger and establishing a synergistic “ferroptosis–inflammation (macrophage infiltration)–fibrosis (TGF-β1-mediated)” injury network. The ferroptosis inhibitor Fer-1 and PM confer protection by correcting iron dysregulation and attenuating oxidative stress. (4) GPX4 deficiency and iron overload. GPX4 loss relieves Bach1-mediated transcriptional repression, upregulating HO-1 expression and promoting heme degradation to release Fe^2+^. Concurrently, LTCCs mediate extracellular Fe^2+^ influx, while SLC40A1-mediated iron efflux is impaired. These three events cooperatively expand the labile iron pool (LIP). LIP-derived Fe^2+^ catalyzes hydroxyl radical (·OH) generation via the Fenton reaction, attacking membrane PUFAs and initiating lipid peroxidation chain reactions. (5) p53-Park7 axis. DOX promotes Park7 degradation in a p53-dependent manner, impairing iron–sulfur cluster synthesis and inactivating aconitase. Inactive aconitase converts to IRP1, which enters the nucleus, upregulating TfR1 and downregulating ferritin expression, causing cytosolic and mitochondrial iron accumulation. (6) Additional regulators. STAT3 serves as a critical modulator of ferroptosis.

In summary, iron chelators suppress ferroptosis by reducing the labile iron pool in cardiomyocytes. Their capacity to improve cardiac function and survival outcomes is well established in iron overload-associated cardiomyopathy. Future efforts should focus on defining indications for iron chelation therapy specifically in DCM, optimizing dosing regimens, evaluating long-term safety, and conducting DCM-specific clinical trials. Table 6 summarizes the intervention targets, experimental models, and evidence types of ferroptosis. Figure 6 depicts ferroptosis signaling in dilated cardiomyopathy.

**TABLE 6:** Ferroptosis-related targets and therapeutic strategies in DCM with corresponding experimental evidence.

Ferroptosis intervention target	Therapeutic strategy or drug	Brief description of core mechanism	Experimental model/evidence type
GPX4 (Jiang et al., 2021; Guo X et al., 2025)	Supplementation or activation of GPX4	Enhances the reduction of phospholipid hydroperoxides, directly blocking ferroptosis execution	Cardiomyocyte-specific *GPX4* knockout mouse model
Iron overload (Pennell et al., 2006; Cooray et al., 2018)	Iron chelators (deferoxamine, deferiprone, and deferasirox)	Reduces the labile iron pool, inhibiting the Fenton reaction and lipid peroxidation	Clinical studies in β-thalassemia and transfusion-related iron overload patients
Iron overload (Kolnagou et al., 2008)	Deferiprone (>80 mg/kg/day) combined with deferoxamine	Synergistically clears myocardial iron via a “shuttling” mechanism, improving cardiac function	Randomized controlled trial in β-thalassemia patients with heart failure
Iron overload (Porter et al., 2013)	Deferiprone combined with N-acetylcysteine	Reduces cardiac iron deposition, improves mitochondrial function, and enhances cardioprotection	Iron-overloaded rat model
NFS1 (She et al., 2025)	AAV9-mediated *NFS1* overexpression	Restores iron-sulfur cluster synthesis, blocks the “iron starvation response,” and reverses mitochondrial iron accumulation	Mst1-overexpressing DCM mouse model
Ferroptosis (core) (She et al., 2025)	Ferroptosis inhibitor ferrostatin-1 (Fer-1)	Inhibits lipid peroxidation, reverses iron overload, and improves cardiac function in Mst1-overexpressing DCM mice	Mst1-overexpressing DCM mouse model
p53-Park7 axis (Pan et al., 2023)	*p53* knockout or Park7 overexpression	Restores iron-sulfur cluster activity and iron homeostasis, suppressing DOX-induced ferroptosis	DOX-induced DCM cellular and mouse models
Blockade of “ferroptosis-inflammation” feedback loop (Haesen et al., 2024)	Pyridoxamine (vitamin B6 derivative)	Restores redox and iron metabolic homeostasis, inhibits ferroptosis and TGF-β1-mediated fibrosis	DOX-induced DCM rat model
m^6^A modification (Zhang et al., 2025)	Targeting m^6^A regulatory enzymes (METTL3/METTL14)	Modulates *SLC7A11* expression, balances cell death and survival, and improves mitochondrial function	Lipopolysaccharide (LPS)-induced cardiomyocyte model
lncRNA-ZFAS1 (Ni et al., 2021)	Inhibition of lncRNA-ZFAS1	Relieves miR-150-5p sponging, upregulates CCND2, and suppresses cardiomyocyte ferroptosis	Diabetic cardiomyopathy mouse model/cellular assays
circPIK3C2A (Miao et al., 2020)	Targeting the circPIK3C2A/miR-31-5p/TFRC axis	Blocks circPIK3C2A-mediated sponging of miR-31-5p, reduces TFRC expression, and alleviates iron overload	Iron-overload myocardial injury mouse model
NCOA4 (Feng et al., 2024)	NCOA4 knockdown	Inhibits ferritinophagy, reduces free iron release, and attenuates lipid peroxidation	Pressure-overload heart failure mouse model
mTOR (Ito et al., 2021)	Moderate mTOR inhibition	Restores autophagic homeostasis and alleviates ferroptosis and myocardial injury (excessive inhibition must be avoided)	Cardiomyocyte-specific *mTOR* knockout mouse model
OTUD1 (Duan et al., 2024)	Modulation of OTUD1 expression	Regulates B cells and dendritic cells in the immune microenvironment, influencing ferroptosis-related immune heterogeneity	Bioinformatic analysis of DCM patients
STAT3 (Wang Z. et al., 2022)	Targeting STAT3	Serves as a core ferroptosis regulator in DCM; modulating its activity influences ferroptotic progression	Comparative bioinformatic analysis of DCM and HCM
SLC22A5/OCTN2 (Loos et al., 2023)	Restoration of carnitine transport	Corrects lipid accumulation and metabolic remodeling, thereby suppressing ferroptosis	Genetic model of primary carnitine deficiency-associated DCM

## 7 Cuproptosis in DCM

Copper is an essential trace element. Its ions exhibit potent redox activity through interactions with diverse proteins and enzymes, functioning as cofactors for numerous indispensable enzymatic reactions (Lutsenko et al., 2025). Cuproptosis is a recently identified PCD modality. Copper ions accumulate within mitochondria and bind to lipoylated proteins of the tricarboxylic acid (TCA) cycle, driving aggregation of these lipoylated moieties. This process precipitates the loss of iron–sulfur cluster proteins, elicits proteotoxic stress, and ultimately culminates in cell death (Tsvetkov et al., 2022).

Following cellular entry via the copper transporter solute carrier family 31 member 1 (SLC31A1) or copper ionophores, cupric ions (Cu^2+^) specifically bind dihydrolipoamide S-acetyltransferase (DLAT), a lipoylated enzyme central to the TCA cycle. This interaction destabilizes iron–sulfur (Fe–S) cluster proteins and precipitates mitochondrial proteotoxic stress, conferring a gain-of-function toxicity on DLAT. Mechanistically, copper-dependent oligomerization of lipoylated DLAT drives aberrant aggregation that culminates in cell death. Intracellular copper homeostasis is jointly governed by the copper importer SLC31A1 and the copper exporter ATPase copper transporting beta (ATP7B). Ferredoxin 1 (FDX1), a critical copper reductase, reduces Cu^2+^ to the more reactive cuprous ion (Cu^+^) while concurrently facilitating the degradation of Fe–S cluster proteins (Wang et al., 2024). Furthermore, copper-induced damage to mitochondrial respiratory chain complexes activates the energy sensor AMP-activated protein kinase (AMPK), thereby accelerating cuproptosis and promoting the release of the pro-inflammatory mediator high mobility group box 1 (HMGB1) (Wang et al., 2024).

Huo et al. (2023) provided the first direct functional evidence for cuproptosis in a diabetic cardiomyopathy model. Advanced glycation end products (AGEs) transcriptionally upregulate the copper importer SLC31A1 by activating transcription factor 3 (ATF3) and spleen focus-forming virus proviral integration oncogene (SPI1). This drives copper accumulation in cardiomyocytes; loss of Fe-S cluster proteins, including FDX1, lipoic acid synthetase (LIAS), NADH:ubiquinone oxidoreductase core subunit S8 (NDUFS8), and aconitase 2 (ACO2); and reduced lipoylation of DLAT, accompanied by impaired mitochondrial respiratory function. Protein and mRNA expression profiles in diabetic mouse hearts align with cuproptotic signatures, and cell death induced by copper ionophores is selectively rescued by copper chelators. These findings establish the pathogenic AGE–ATF3/SPI1–SLC31A1–cuproptosis axis in diabetic myocardial injury and provide the first complete evidence chain encompassing signal regulation, execution events, and functional consequences for cuproptosis in diabetic cardiomyopathy (Huo et al., 2023).

Notably, the association between diabetic cardiomyopathy and disrupted copper homeostasis attracted attention well before the conceptualization of cuproptosis. A series of studies revealed that the myocardium of diabetic rats exhibits copper deficiency and defective cellular copper trafficking. These abnormalities include reduced expression of the high-affinity copper transporter 1 (CTR1), impaired copper delivery from the copper chaperone for superoxide dismutase (CCS) to superoxide dismutase 1 (SOD1), and diminished metallothionein levels. Such defects correlate closely with left ventricular dysfunction (Zhang et al., 2020). Mechanistically, diabetes-induced myocardial copper trafficking defects further provoke mitochondrial damage and dysregulated energy metabolism (Jüllig et al., 2007). Treatment with the Cu(II)-selective chelator triethylenetetramine (TETA) restores the expression and function of myocardial copper transporters, repairs mitochondrial cuproenzyme activity, and normalizes both myocardial copper levels and cardiac function in diabetic rats (Zhang et al., 2014). These observations underscore the central role of disrupted myocardial copper homeostasis in diabetic cardiomyopathy. The generalizability of these findings to primary DCM and their direct mechanistic link to the cuproptosis pathway await further validation.

Although elevated copper concentrations are significantly associated with the onset of heart failure and poor prognosis in DCM (Zhang et al., 2022), mechanistic studies directly addressing cuproptosis in DCM are lacking. In light of this gap, Bian et al. (2023) postulated that cuproptosis may also contribute to DCM pathogenesis. Employing machine learning algorithms to interrogate the relationship between DCM-associated genes and cuproptosis, they predicted targets for herbal intervention. Two signature genes, *FDX1* and *SLC31A1*, performed robustly in external validation datasets. Molecular docking models indicated that the natural compounds rutin and polydatin bind favorably to *FDX1* and *SLC31A1*, respectively, suggesting potential therapeutic modulation of cuproptosis in DCM through these targets.

Bioinformatic analyses have explored the genetic links between cuproptosis and DCM, identifying key gene modules common to both. Six signature genes, *septin 1* (*SEPTIN1*), *C-type lectin domain family 11 member A* (*CLEC11A*), *ISG15 ubiquitin-like modifier* (*ISG15*), *prolyl 3-hydroxylase 3* (*P3H3*), *serine dehydratase-like* (*SDSL*), and *INKA1* (*official full name INKA1*), were identified as potential novel diagnostic biomarkers for DCM. Expression of these genes correlates closely with immune cell infiltration, implicating cuproptosis in the immunoregulatory processes underlying DCM (Lin et al., 2024).

The evidence summarized above, however, derives exclusively from bioinformatic predictions and correlative analyses. Whether cuproptosis genuinely occurs in the DCM myocardium, whether core execution events such as DLAT oligomerization and Fe–S cluster protein degradation are operative, and whether FDX1, SLC31A1, or the six signature genes truly govern the cuproptotic pathway all lack direct functional experimental validation.

*In vitro*, liposomal delivery of a microRNA-185-5p inhibitor (Lipo@miR-185-5p inhibitor) markedly attenuates both apoptosis and cuproptosis in H9C2 cells and induced pluripotent stem cell-derived cardiomyocytes. *In vivo*, this targeted liposomal formulation significantly improves cardiac function in DOX-induced DCM mice. These findings implicate the inhibitor-loaded targeted delivery system as a potential therapeutic strategy for DCM (Xu et al., 2025).

Although these investigations have established preliminary associations between cuproptosis-related genes and DCM, suggesting potential therapeutic value in targeting this pathway, the available evidence remains confined to bioinformatic predictions and indirect inferences derived from altered biomarker expression. Direct functional validation and mechanistic dissection of cuproptosis in DCM are virtually absent. First, direct functional evidence for cuproptosis in DCM is lacking. Existing studies rely exclusively on bioinformatic predictions or surrogate marker changes. No investigation has directly validated whether cuproptosis genuinely occurs in DCM animal models or patient myocardial samples, assessed its magnitude, or determined whether it constitutes a significant contributor to cardiomyocyte loss. Core execution events such as DLAT oligomerization and Fe–S cluster protein degradation have never been directly detected in DCM. Second, the causal chain linking copper dyshomeostasis to cuproptotic execution remains incomplete. Elevated copper levels correlate with adverse DCM outcomes, yet whether high copper invariably drives cardiomyocyte cuproptosis remains unproven. The relative contributions of copper-mediated oxidative stress, mitochondrial respiratory chain damage, and crosstalk with other PCD pathways cannot be distinguished from existing data. Third, a substantial gap persists between bioinformatic prediction and functional validation. Although FDX1, SLC31A1, and the six signature genes were identified through computational screening, their expression dynamics in DCM myocardium, their functional engagement with cuproptotic execution, and the actual interventional efficacy of rutin or polydatin all await experimental verification. Fourth, the interplay between cuproptosis and other PCD modalities remains unknown. The capacity of a miR-185-5p inhibitor to concurrently suppress markers of apoptosis and cuproptosis implies shared upstream regulatory signals or execution-level crosstalk. The specific molecular connection points, relative contributions, and relationships across distinct DCM subtypes are entirely undefined. Fifth, the specific drivers of cuproptosis in DCM have not been identified. Cuproptosis is documented in diabetic cardiomyopathy, yet DCM encompasses a broad etiological spectrum (genetic, inflammatory, and drug-induced). Whether differences exist among subtypes regarding triggers of copper dysregulation, cuproptosis activation, signaling preferences, or pathological weighting remains wholly unexplored.

**FIGURE 7:**
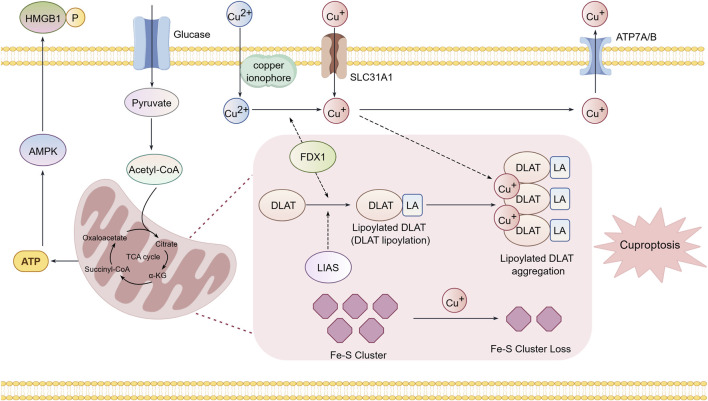
Core signaling of cuproptosis. Intracellular Cu^2+^ enters via transporter SLC31A1 and specifically binds the lipoylated tricarboxylic acid cycle enzyme DLAT, driving its copper-dependent aberrant oligomerization and destabilizing iron-sulfur cluster proteins. FDX1 acts as a critical reductase, converting Cu^2+^ to Cu^+^ while exacerbating iron–sulfur cluster degradation, collectively precipitating mitochondrial proteotoxic stress. This process also damages the mitochondrial respiratory chain, which activates the energy sensor AMPK and promoting the release of the damage-associated molecule HMGB1, thereby accelerating cuproptosis. Intracellular copper homeostasis is maintained through the coordinated actions of the importer SLC31A1 and the exporter ATP7B.

In summary, the investigation of cuproptosis in DCM remains in its infancy. Priority should be directed toward the following objectives: (i) direct detection of core cuproptotic events (DLAT oligomerization, Fe–S cluster protein abundance) in DCM animal models and patient myocardial samples; (ii) establishing a causal chain linking disrupted copper metabolism, cuproptotic execution, and the DCM phenotype; (iii) delineating the relative contribution of cuproptosis *versus* other copper-induced cytotoxic effects; (iv) resolving the reciprocal regulatory networks connecting cuproptosis with apoptosis, necroptosis, pyroptosis, and autophagy; and (v) validating the true functional roles of bioinformatically predicted targets in DCM-associated cuproptosis. The translational potential of cuproptosis as a therapeutic target in DCM hinges on a decisive shift from correlative inference to rigorous functional validation. Table 7 summarizes the intervention targets, experimental models, and evidence types of cuproptosis. Figure 7 depicts the core signaling pathways of cuproptosis.

**TABLE 7:** Cuproptosis-targeting agents and biomarkers in DCM.

Cuproptosis target	Therapeutic/diagnostic strategy or agent	Brief description of core mechanism	Experimental model/evidence type
Myocardial copper trafficking defect (Zhang et al., 2020; Jüllig et al., 2007; Zhang et al., 2014)	Cu(II)-selective chelator triethylenetetramine (TETA)	Restores expression and function of myocardial copper transporters (CTR1/CCS), repairs mitochondrial cuproenzyme activity, and reverses copper deficiency and cardiac dysfunction	Diabetic cardiomyopathy rat model (streptozotocin-induced)
ATF3/SPI1-SLC31A1 axis (Huo et al., 2023)	Copper chelator tetrathiomolybdate	AGEs upregulate SLC31A1 transcription *via* ATF3 and SPI1, increasing copper influx and causing loss of Fe-S cluster proteins (FDX1, LIAS, NDUFS8, and ACO2) and reduced DLAT lipoylation to trigger cuproptosis; copper chelator provides specific rescue	AGE-treated AC16 human cardiomyocyte cell line plus STZ-induced diabetic mice and db/db diabetic mice
SEPTIN1, CLEC11A, ISG15, P3H3, SDSL, and INKA1 (Lin et al., 2024)	Diagnostic biomarker screening	WGCNA and LASSO algorithms identified six signature genes linked to both DCM and cuproptosis; their expression correlates with immune cell infiltration, suggesting that cuproptosis may participate in DCM *via* immune regulation	GEO DCM datasets (GSE141910 and GSE17800) plus bioinformatic analyses (differential expression, WGCNA, LASSO regression, and immune infiltration)
FDX1 (predicted) (Bian et al., 2023)	Rutin	Molecular docking indicates high-affinity binding to FDX1; may regulate cuproptosis through FDX1 inhibition	GEO DCM datasets plus machine learning screening plus molecular docking simulations
SLC31A1 (predicted) (Bian et al., 2023)	Polydatin	Molecular docking indicates binding to SLC31A1; may regulate cuproptosis by inhibiting copper uptake	GEO DCM datasets plus machine learning screening plus molecular docking simulations
miR-185-5p (Cheedipudi et al., 2019)	Liposome-encapsulated miR-185-5p inhibitor	Suppresses miR-185-5p, reduces apoptotic and cuproptotic markers, and improves cardiac function and fibrosis	H9C2 cells plus induced pluripotent stem cell-derived cardiomyocytes plus DOX-induced DCM mouse model

## 8 Hierarchical contributions of six PCD pathways in DCM

### 8.1 Direct quantitative comparison of apoptosis and pyroptosis

Apoptosis has long been regarded as the principal mechanism underlying cardiomyocyte loss in DCM. Narula et al. (1996) provided the earliest confirmation of apoptosis in myocardial tissue from patients with end-stage heart failure secondary to DCM, establishing the fundamental concept that PCD participates in DCM pathogenesis. A 2000 study of endomyocardial biopsies from patients with end-stage DCM reported an average TUNEL-positive cardiomyocyte percentage of approximately 4% (Kavantzas et al., 2000). DNA fragmentation detected by TUNEL, however, is not exclusive to apoptosis; pyroptotic cells also exhibit TUNEL positivity (Zeng et al., 2020). Early studies relying solely on TUNEL staining may, therefore, not have accurately distinguished apoptosis from other PCD forms, and their quantitative findings warrant cautious interpretation.

Zeng et al. (2020) performed the first direct quantitative comparison of pyroptosis and apoptosis in myocardial tissue from patients with idiopathic DCM. Analysis of samples from nine patients revealed that (i) abundant pyroptotic cells were present in all cases, with the majority identified as cardiomyocytes (69.20% ± 5.04% *versus* 30.80% ± 5.04% non-cardiomyocytes); (ii) the percentage of pyroptotic cardiomyocytes (11.62% ± 5.30%) significantly exceeded that of apoptotic cardiomyocytes (7.57% ± 2.86%); and (iii) the pyroptotic cardiomyocyte percentage tended to correlate inversely with left ventricular ejection fraction. Supporting animal experiments further demonstrated that knockout of *NLRP3* or *CASP1* suppresses pyroptosis, substantially attenuates myocardial pathology, and improves cardiac function. This remains the sole study to date offering a direct quantitative comparison of these two PCD forms in human DCM myocardium, suggesting that in idiopathic DCM the contribution of cardiomyocyte pyroptosis may surpass that of apoptosis.

### 8.2 Reassessing the role of apoptosis

The observation that pyroptosis outnumbers apoptosis in quantitative comparisons does not diminish the significance of apoptosis in DCM. Apoptosis remains the most extensively studied and mechanistically best-characterized PCD form in this disease. TTNtv, which accounts for approximately 25% of familial DCM, directly participates in genetic DCM pathogenesis by modulating the NF-κB–IER3 axis to regulate expression of the protective factors Bcl-2, Bcl-2L1, and AKT1 (Zhou et al., 2017). In *LMNA*-related DCM, pathogenic activation of the E2F/TP53 pathway likewise directly induces cardiomyocyte apoptosis (Chen et al., 2019). Moreover, in DOX-induced DCM, multiple signaling cascades, including the AKT/p38 MAPK and Hsp27/p53 axes, converge on apoptotic execution (Kanagasabai et al., 2021; Xuan et al., 2020). Apoptosis may therefore retain a dominant role in genetic and drug-induced DCM, whereas the prominent contribution of pyroptosis is likely more pronounced in idiopathic or inflammatory DCM subtypes.

### 8.3 Relative contributions of apoptosis, necroptosis, and autophagy

Activation of necroptosis in DCM is supported by both clinical and animal model evidence. Corsetti et al. (2019) analyzed myocardial tissue from 22 DCM patients undergoing cardiac transplantation and observed that progressive autophagy can trigger secondary cell death, with necroptosis and oncosis occurring more frequently than apoptosis. Wu Y. et al. (2021) demonstrated in an EAM rat model that apoptosis, necroptosis, and autophagy each contribute significantly to cardiomyocyte death. Inhibitors targeting each of these three PCD pathways (Nec-1, 3-MA, and zVAD-fmk) independently attenuated myocardial inflammation and improved cardiac function, indicating distinct contributions of each pathway to myocardial injury. Direct quantitative data regarding the contributions of necroptosis and autophagy in DCM, however, are lacking. Systematic comparisons of their relative weights across different DCM etiological subtypes have yet to be performed.

### 8.4 Evidence hierarchy for ferroptosis and cuproptosis

Investigations of ferroptosis in DCM have largely centered on bioinformatic predictions and indirect alterations in surrogate markers. Direct evidence for core ferroptotic execution events in DCM patient myocardium or animal models is absent. FDX1 and SLC31A1 were identified as signature genes (Bian et al., 2023), while six diagnostic biomarkers (e.g., SEPTIN1) linked to immune infiltration were also reported (Lin et al., 2024), and a miR-185-5p inhibitor concurrently reduces markers of apoptosis and cuproptosis (Xu et al., 2025). Core cuproptotic events, such as DLAT oligomerization and Fe–S cluster protein degradation, however, have never been directly detected in DCM. The actual quantitative contributions of ferroptosis and cuproptosis in DCM, therefore, lack functional evidentiary support, positioning these two modalities as the least substantiated among the six PCD forms.

### 8.5 Implications of the PANoptosis concept

The recent emergence of the PANoptosis concept offers a fresh perspective for understanding the hierarchical relationships among PCD pathways. Bi et al. (2022) observed that FUN14 domain containing 1 (FUNDC1) is downregulated in heart tissue from DCM patients and DOX-treated mice and that FUNDC1 deficiency exacerbates DOX-induced mitochondrial damage and cardiomyocyte PANoptosis. Olcum et al. (2023a) identified PANoptosis as a prominent phenotypic feature in desmoplakin cardiomyopathy and demonstrated that β-catenin inactivation mitigates PANoptosis. The existence of PANoptosis suggests that cardiomyocytes in DCM may simultaneously engage multiple PCD execution pathways rather than operating through a single modality in isolation, underscoring an integrated regulatory model characterized by synergistic activation of multiple pathways.

## 9 Discussion

Multiple PCD pathways in DCM do not function in isolation. Rather, they converge on shared signaling nodes to establish a complex crosstalk network (Bertheloot et al., 2021; Frank and Vince, 2019; Zheng and Kanneganti, 2020; Roberts et al., 2022; Guo et al., 2016).

### 9.1 Interplay between apoptosis and necroptosis

Both modalities share upstream signals originating from TNFR engagement. RIPK1 operates as a central hub: its association with FADD and caspase-8 drives apoptosis, whereas its interaction with RIPK3 and MLKL initiates necroptosis (Yin et al., 2022; Fritsch et al., 2019; Pasparakis and Vandenabeele, 2015; Geng et al., 2017). TAB2 serves as an adaptor linking TNFR1 to TAK1 (Takaesu et al., 2000). Yin et al. (2022) observed that TAB2-deficient mice develop a DCM-like phenotype accompanied by markedly elevated myocardial RIPK1, RIPK3, and MLKL. Mechanistically, TAB2 suppresses both apoptosis and necroptosis in a manner independent of NF-κB but strictly dependent on RIPK1. Inactivation of RIPK1 reverses the cardiomyocyte loss precipitated by TAB2 deficiency. These findings establish the TAB2–TAK1–RIPK1 axis as a pivotal protective signal governing both PCD modalities. The expression profile of this axis in human DCM and its potential crosstalk with NF-κB signaling, however, await further elucidation.

### 9.2 Interplay between autophagy and apoptosis

Zhang S. et al. (2023) demonstrated that DOX suppresses autophagy to induce cardiomyocyte apoptosis, a requisite step in DCM pathogenesis. Activation of AMPK and consequent downregulation of mTOR markedly attenuate DOX-induced oxidative stress, preserve autophagic flux, and reduce cardiomyocyte apoptosis (Zhang S. et al., 2023). F-box protein 32 (FBXO32), a component of the cardiac-specific E3 ubiquitin ligase complex, harbors recessive variants linked to familial DCM (Adams et al., 2007; Al-Yacoub et al., 2016). FBXO32 variants inhibit SCF complex activity, impair selective autophagic clearance, and simultaneously provoke ER stress dysregulation characterized by CHOP and ATF2 upregulation, caspase-3 activation, and markedly increased apoptosis (Al-Yacoub et al., 2021). FBXO32 variants, therefore, drive genetic DCM through at least two convergent mechanisms: impaired autophagy and apoptotic activation. Cardiomyocytes rely on basal autophagy for homeostasis (Ikeda et al., 2022), and autophagy suppresses apoptosis partly through the clearance of damaged mitochondria (Nikoletopoulou et al., 2013). When autophagy is severely compromised, cellular stress tolerance declines, precipitating irreversible cell loss that cumulatively progresses to DCM (Zou and Xie, 2013). The direct molecular connection linking FBXO32 variant-induced autophagic impairment to apoptotic activation remains undefined.

Potential crosstalk among other PCD modalities. Several crosstalk relationships established in other disease models remain unverified in DCM and may represent avenues for future investigation. First, MLKL-mediated membrane damage and potassium efflux during necroptosis can activate the NLRP3 inflammasome, implying interaction between necroptosis and pyroptosis (Bertheloot et al., 2021; Frank and Vince, 2019). Second, ROS-induced lipid peroxidation plays a critical role in apoptosis, autophagy, and ferroptosis (Su et al., 2019). Third, the TCA cycle provides ROS that fuel ferroptosis (Yue et al., 2023), and the core cuproptosis substrates, lipoylated proteins, likewise derive from the TCA cycle, increasing the possibility that cuproptosis and ferroptosis may crosstalk through this shared metabolic hub (Tsvetkov et al., 2022). Notably, these proposed interactions currently lack direct experimental validation in DCM models.

Current evidence consistently demonstrates that six PCD modalities, namely, apoptosis, necroptosis, autophagy, pyroptosis, ferroptosis, and cuproptosis, participate in DCM pathogenesis. These findings derive from diverse sources, including explanted hearts from DCM transplant recipients, transgenic and drug-induced DCM models in mice and rats, zebrafish, and *in vitro* cardiomyocyte systems. PCD crosstalk research, however, faces considerable challenges. First, studies directly validating PCD interactions in DCM models remain exceedingly sparse; most proposed crosstalk relationships are extrapolated from findings in other disease contexts. Second, existing investigations predominantly focus on unidirectional regulation between two pathways and lack systematic resolution of hierarchical multi-pathway network relationships. Third, the roles of emerging PCD forms, including mitotic death, immunogenic cell death, and poly (ADP-ribose) polymerase 1-dependent parthanatos, in DCM remain entirely unexplored. Future efforts should systematically map PCD crosstalk networks across distinct etiological DCM models and disease stages and define the regulatory logic at critical convergence nodes, thereby establishing a mechanistic foundation for rational multi-pathway targeted combination therapy.
